# Cardiovascular Toxicities Secondary to Biotherapy and Molecular Targeted Therapies in Neuroendocrine Neoplasms: A Systematic Review and Meta-Analysis of Randomized Placebo-Controlled Trials

**DOI:** 10.3390/cancers13092159

**Published:** 2021-04-30

**Authors:** Charalampos Aktypis, Maria-Eleni Spei, Maria Yavropoulou, Göran Wallin, Anna Koumarianou, Gregory Kaltsas, Eva Kassi, Kosmas Daskalakis

**Affiliations:** 1 Department of Gastroenterology, Laiko General Hospital, Medical School of National & Kapodistrian University, 11527 Athens, Greece; aktypischar@live.com; 2 1st Department of Propaedeutic Internal Medicine, Endocrine Unit, National and Kapodistrian, University of Athens, 11527 Athens, Greece; marilena_0108@hotmail.com (M.-E.S.); maria.yavropoulou.my@gmail.com (M.Y.); gkaltsas@endo.gr (G.K.); evakassis@gmail.com (E.K.); 3 Department of Surgery, Faculty of Medicine and Health, Örebro University, 701 85 Örebro, Sweden; goran.wallin@regionorebrolan.se; 4 Hematology-Oncology Unit, Fourth Department of Internal Medicine, Attikon Hospital, Medical School, National and Kapodistrian University of Athens, 124 62 Athens, Greece; akoumari@yahoo.com; 5 Department of Biological Chemistry, Medical School, National and Kapodistrian University of Athens, 11527 Athens, Greece

**Keywords:** neuroendocrine neoplasms, molecular targeted therapies, mTOR inhibitors, somatostatin analogs, TPH inhibitors, meta-analysis

## Abstract

**Simple Summary:**

Recent innovations in molecular pathogenesis of neuroendocrine neoplasms (NEN) and improvements in their multidisciplinary management, including the introduction of novel targeted therapies have contributed to favorable patient outcomes. Compared with traditional chemotherapy, targeted therapies have fewer toxicities and a more distinct safety profile. However, treatment-induced cardiovascular toxicities are occasionally critical issues in NEN management. Herein, we present a comprehensive summary of high quality randomized evidence with the methodology of a systematic review and quantitative meta-analysis on the safety profile of biotherapy and molecular targeted therapies in advanced and/or metastatic NEN with a special focus on cardiovascular toxicities in order to promote a patient-tailored approach and assist clinicians involved in the management of NEN patients.

**Abstract:**

A broad spectrum of novel targeted therapies with prime antitumor activity and/or ample control of hormonal symptoms together with an overall acceptable safety profile have emerged for patients with metastatic neuroendocrine neoplasms (NENs). In this systematic review and quantitative meta-analysis, the PubMed, EMBASE, Cochrane Central Register of Controlled Trials and clinicaltrials.gov databases were searched to assess and compare the safety profile of NEN treatments with special focus on the cardiovascular adverse effects of biotherapy and molecular targeted therapies (MTTs). Quality/risk of bias were assessed using GRADE criteria. Placebo-controlled randomized clinical trials (RCTs) in patients with metastatic NENs, including medullary thyroid cancer (MTC) were included. A total of 3695 articles and 122 clinical trials registered in clinicaltrials.gov were screened. We included sixteen relevant RCTs comprising 3408 unique patients assigned to different treatments compared with placebo. All the included studies had a low risk of bias. We identified four drug therapies for NENs with eligible placebo-controlled RCTs: somatostatin analogs (SSAs), tryptophan hydroxylase (TPH) inhibitors, mTOR inhibitors and tyrosine kinase inhibitors (TKI). Grade 3 and 4 adverse effects (AE) were more often encountered in patients treated with mTOR inhibitors and TKI (odds ratio [OR]: 2.42, 95% CI: 1.87–3.12 and OR: 3.41, 95% CI: 1.46–7.96, respectively) as compared to SSAs (OR:0.77, 95% CI: 0.47–1.27) and TPH inhibitors (OR:0.77, 95% CI: 0.35–1.69). MTOR inhibitors had the highest risk for serious cardiac AE (OR:3.28, 95% CI: 1.66–6.48) followed by TKIs (OR:1.51, 95% CI: 0.59–3.83). Serious vascular AE were more often encountered in NEN patients treated with mTOR inhibitors (OR: 1.72, 95% CI: 0.64–4.64) and TKIs (OR:1.64, 95% CI: 0.35–7.78). Finally, patients on TKIs were at higher risk for new-onset or exacerbation of pre-existing hypertension (OR:3.31, 95% CI: 1.87–5.86). In conclusion, SSAs and TPH inhibitors appear to be safer as compared to mTOR inhibitors and TKIs with regards to their overall toxicity profile, and cardiovascular toxicities in particular. Special consideration should be given to a patient-tailored approach with anticipated toxicities of targeted NEN treatments together with assessment of cardiovascular comorbidities, assisting clinicians in treatment selection and early recognition/management of cardiovascular toxicities. This approach could improve patient compliance and preserve cardiovascular health and overall quality of life.

## 1. Introduction

Neuroendocrine neoplasms (NENs) comprise a group of diverse histopathological entities across different organs and systems, including the gastrointestinal system, the lungs, the adrenals and the thyroid. Although the majority of NENs are well differentiated (WD) and may exhibit a prolonged indolent course, some patients categories, e.g., the ones with medullary thyroid cancer (MTC), atypical lung carcinoids (LCs) and pancreatic NENs of higher proliferation may exhibit a more aggressive course [1,2]. Many NEN patients are diagnosed with established distant metastases or exhibit progress to stage IV under disease surveillance [3]. Systemic cytotoxic chemotherapy has a generally low response rate in patients with metastatic WD NENs of lower proliferation and MTC and is nevertheless associated with serious toxicities. On the other hand, prime anti-tumor activity and/or control of hormonal excess syndromes has been demonstrated for targeted agents in NENs, resulting in the approval of somatostatin analogs (SSAs) and tryptophan hydroxylase (TPH) inhibitors for gastroenteropancreatic NENs, also referred to as biotherapy, as well as novel molecular targeted therapies (MTTs), such as the mTOR inhibitor everolimus and a number of tyrosine kinase inhibitors (TKIs) with the latter being approved across a diverse spectrum of NEN primaries, including pancreatic NENs and MTC [4,5].

In the last decades, an increment in the prevalence of NENs along with a prolongation in life expectancy of these patients has been observed despite a rising prevalence of cardiovascular diseases in the elderly [1]. On the other hand, carcinoid heart disease, a rare cardiac manifestation involving the right-sided heart valves, constitutes a well-recognized sequela in patients with small intestinal neuroendocrine neoplasms (SI-NENs) often complicating the disease clinical course and eventually leading to right heart failure [6]. Finally, NEN metastases to the heart are rare, with associated clinical features ranging from asymptomatic patients to heart failure [7]. All these factors taken together with the cardiovascular side effects of different agents in the therapeutic NEN armamentarium may have a negative impact on patient outcomes, including quality of life and possibly survival outcomes. Nevertheless, the continual and occasionally prolonged nature of the administration of targeted agents leads to new challenges in their application with respect to the management of anticipated cardiovascular toxicities [5,8].

The most frequent side effects of SSAs consist mainly of gastrointestinal toxicities, with potentially a beneficial effect on cardiac parameters in the setting of acromegalic heart disease, a constellation of cardiac complications associated with acromegaly that involves nearly all aspects of the cardiovascular system [9]. In addition, SSAs and TPH inhibitors inhibit serotonin secretion from the tumor and subsequently lower 5-HIAA levels, relieving carcinoid syndrome, a rare secretory syndrome mainly associated with small intestinal and bronchial NENs that becomes manifest when serotonin and other vasoactive substances from the tumor enter the systemic circulation escaping hepatic degradation [4]. However, SSAs do not unequivocally reverse the progression of the carcinoid cardiac involvement nor improve survival in the setting of carcinoid heart disease [10]. The role of the recently introduced telotristat ethyl for prevention or control of carcinoid heart disease remains largely unknown, but could be elucidated in the near future as we obtain further evidence from clinical trials.

With regards to pathophysiology, MTTs may induce cardiovascular adverse effects (AEs) as a result of “on-target” and “off-target” mechanisms [11,12]. The on-target toxicity mechanism implicates mainly the mTOR complex 1 pathway with the target of MTT playing a crucial role in oncogenesis and angiogenesis, but also in hypertrophic response and survival of cardiomyocytes [12]. Off-target toxicity on the other hand, implicates an unintentional inhibition of a kinase that is also important for cardiac cell survival or function. For example certain TKIs induce a cardiomyocyte damage-related lactate dehydrogenase (LDH) release, that is in turn associated with the binding specificity of TKIs to their molecular target [13].

The placebo-controlled RCTs on biotherapy and MTTs for NENs and MTC report treatment-related toxicities according to the National Cancer Institute Common Terminology Criteria for Adverse Events, and therefore constitute a complete resource of treatment-related toxicities [14,15,16]. The present overview provides a comprehensive summary of high quality randomized evidence with the methodology of a systematic review and quantitative meta-analysis on the distinct safety profile of biotherapy and MTTs in advanced and/or metastatic NEN with a special focus on cardiovascular toxicities in order to assist clinicians involved in the management of NEN patients.

## 2. Results

### 2.1. Study Selection

**Figure 1:**
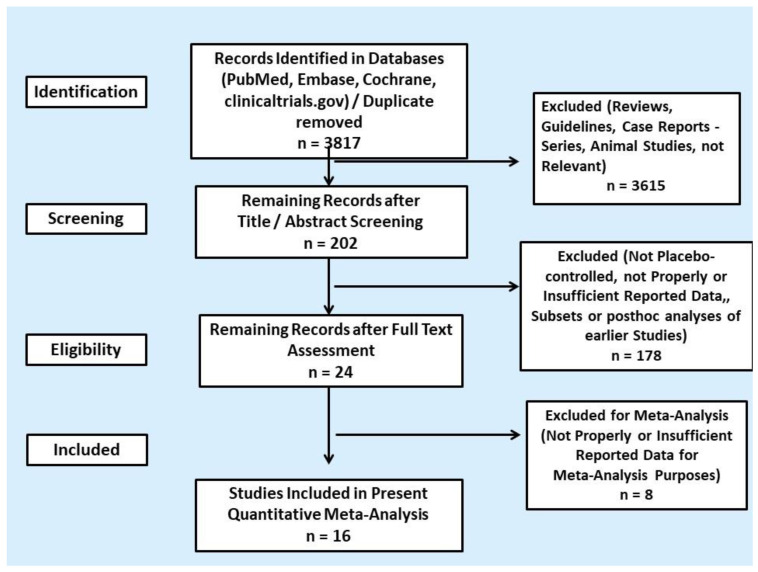
PRISMA flowchart of search results.

We screened 3695 titles and abstracts from PubMed, Excerpta Medica database (EMBASE), Cochrane Central Register of Controlled Trials and additional 122 clinical trials in clinicaltrials.gov and identified 202 potentially eligible RCT reports (Figure 1). Some of the RCTs were reported in multiple publications or were posthoc analysis of the initial RCT; thus, they were excluded. After full article assessment, we included a total of 16 placebo-controlled RCTs reporting cardiovascular toxicities in the quantitative meta-analysis. Only patients with metastatic NET or MTC were included. The results on the safety profile of most RCTs were available from the published article or abstract and clinicaltrials.gov. A total of 3408 unique patients were recruited; four different categories of targeted agents were evaluated: SSAs, the TPH inhibitor telotrist etiprate, the mTOR inhibir everolimus and different TKIs. In particular, six RCTs addressed biotherapy (three RCTs on SSAs and three RCTs on telotrist etiprate) and ten RCTs addressed MTTs (the RADIANT-2, 3 and 4 trials on everolimus and seven RCTs on TKIs). All RCTs in the quantitative meta-analysis were industry sponsored. Study and patient characteristics are provided in Table 1 and Table S1, respectively.

### 2.2. Representation in International Guidelines

Among the sixteen RCTs included in the present meta-analysis, six out of 14 RCTs on WD-NENs are included in the latest 2017 European Neuroendocrine Tumor Society (ENETS) [4], whereas one study on MTC, the ZETA trial, is included in both the 2015 Society American Thyroid Association (ATA) consensus guidelines [15] and the 2012 European Thyroid Association (ETA) guidelines for MTC (Table 1) [16].

### 2.3. Risk of Bias Assessment

In none of the included studies did we encounter high risk for bias in random sequence generation (selection bias), allocation concealment (selection bias), blinding participants and personnel (performance bias), blinding the outcome assessment (detection bias), incomplete outcome data (attrition bias), and selective reporting (reporting bias) (Table 2).

### 2.4. Serious Toxicities’ Profile

**Figure 2:**
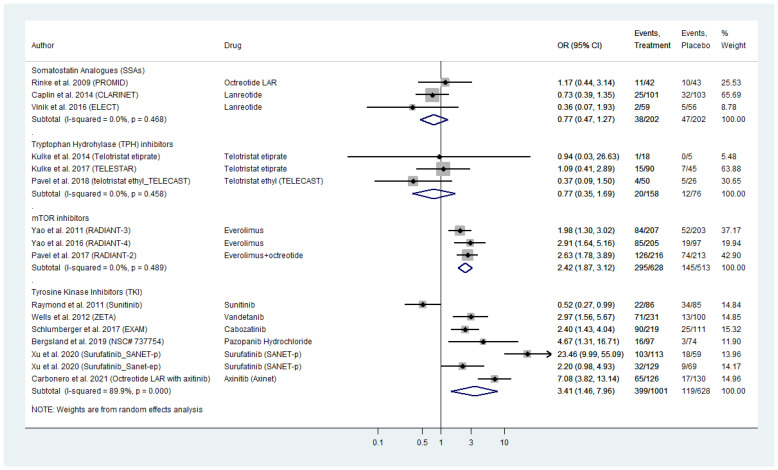
Targeted Agents’ Serious Toxicities profile per drug category in patients with Metastatic Well-Differentiated Neuroendocrine Neoplasms or Medullary Thyroid Cancer (Odds Ratios [OR] with 95% Confidence Intervals). Tyrosine kinase inhibitors exhibited the highest pooled OR: 3.41 (95% CI: 1.46–7.96) followed by everolimus (pooled OR: 2.42 [95% CI: 1.87–3.12]). Somatostatin analogues pooled OR was relatively low( 0.77 [95% CI: 0.47–1.27], same as that of telotristat etiprate (OR: 0.77 [95% CI: 0.35–1.69]).

Sixteen placebo-controlled RCTs compared grade 3 and 4 AE for four different categories of targeted agents in WD-NENs and MTC (Figure 2). TKIs exhibited a pooled odds ratio (OR) for serious AE of 3.41 (95% CI: 1.46–7.96). For surafatinib AE_OR in pancreatic NENs was as high as 23.46 (95% CI: 9.99–55.09), whereas for sunitinib in the same subset of patients AE_OR was 0.52 (95% CI: 0.27–0.99), as compared to placebo. In addition, the recently tested in phase III NEN trials axitinib and pazopanib demontrated a relatively high AE_OR (axitinib AE_OR: 7.08; 95% CI: 3.83–13.14 and pazopanib AE_OR: 4.67; 95% CI: 1.31–16.71, respectively). For MTC, the effect estimates were AE_OR: 2.97 (95% CI, 1.56–5.67) for vandetanib and 2.40 (95% CI: 1.43–4.04) for cabozatinib, as compared to placebo (Figure 2).The mTOR inhibitor everolimus exhibited a pooled OR for serious AE of 2.42 (95% CI: 1.87–3.12). With regards to biotherapy, SSAs demonstrated a pooled OR for serious AE as low as 0.77 (95% CI: 0.47–1.27), which was comparable to that of the TPH inhibitor telotristat etiprate (AE_OR: 0.77; 95% CI: 0.35–1.69; Figure 2). Significant heterogeneity was observed across the subset of studies on TKI (I^2^ = 89.8%, *p*-value < 0.0001; Figure 2). A funnel plot was also produced demonstrating asymmetry (Figure S1A), that was mainly attributed to the recent studies on novel TKIs by Xu et al. on surufatinib in pancreatic NENs and by Carbonero et al. on axitinib in extra-pancreatic NENs (Galbraith’s plot; Figure S1B) [29,30]. Egger’s test showed no indication of publication bias across the included studies (*p*-value > 0.05). In the subset of studies on SSAs, TPH inhibitors and everolimus, we did not observe inter-study heterogeneity or publication bias (Figure 2 and Figure S1A–C).

### 2.5. All Grade Toxicities’ Profile

**Figure 3:**
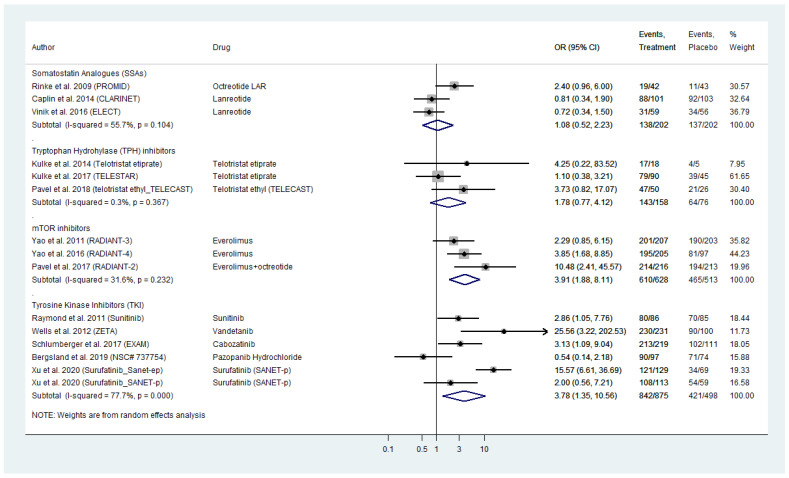
Targeted Agents’ All Grade Toxicities profile per drug category in patients with Metastatic Well-Differentiated Neuroendocrine Neoplasms or Medullary Thyroid Cancer (Odds Ratios with 95% Confidence Intervals). Tyrosine kinase inhibitors (TKI) and everolimus demontrated the highest risk of all grade toxicity (pooled OR: 3.78 [95% CI: 1.35–10.56] and OR: 3.91 [95% CI: 1.88–8.11], respectively).

We conducted a meta-analysis of AE of all grades reported in the included RCTs (Figure 3). Our findings showed comparable figures compared to serious toxicities analysis with TKIs and everolimus demontrating the highest risk of all grade toxicity (pooled TKI AE_OR: 3.78; 95% CI: 1.35–10.56 across both WD-NEN and MTC diagnoses; and pooled everolimus AE_OR: 3.91, 95% CI: 1.88–8.11). SSAs appeared to have the safest all grade toxicity profile (AE_OR: 1.08, 95% CI: 0.52–2.23; Figure 3). 

Significant heterogeneity was evident across the subset of studies on TKI (I^2^ = 77.7%, *p*-value < 0.0001; Figure 3). A funnel plot was demonstrated signs of asymmetry (Figure S2A), that was mainly attributed to the study by Wells et al. on vandetanib in MTC and Xu et al. on surufatinib in pancreatic NENs (Galbraith’s plot; Figure S2B). Egger’s test showed no indication of publication bias across the included studies (*p*-value > 0.05). With regards to SSA, TPH inhibitors and everolimus, we did not observe inter-study heterogeneity or publication bias (Figure 3 and Figure S2A–C).

### 2.6. Serious Cardiac Toxicities’ Profile

**Figure 4:**
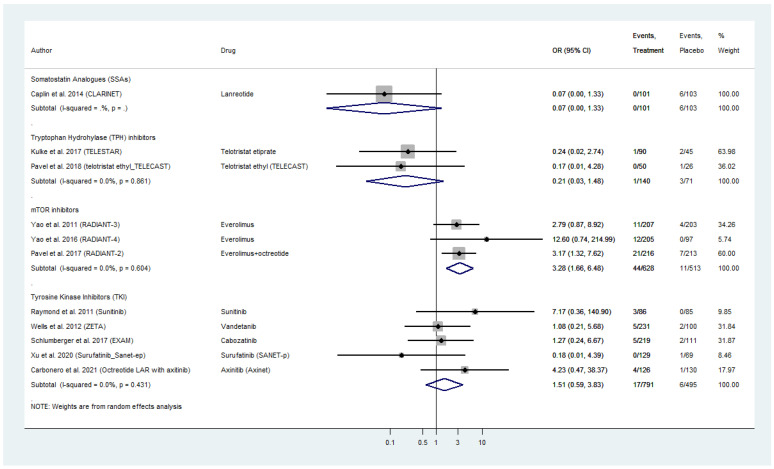
Targeted Agents’ Serious Cardiac Toxicities profile per drug category in patients with Metastatic Well-Differentiated Neuroendocrine Neoplasms or Medullary Thyroid Cancer (Odds Ratios [OR] with 95% Confidence Intervals). Everolimus exhibited the highest pooled OR: 3.28 (95% CI: 1.66–6.48) followed by tyrosine kinase inhibitors with a pooled OR: 1.51 (95% CI: 0.59–3.83). Somatostatin analogs and telotristat etiprate demonstrated a potential prophylactic effect with with an OR: 0.07 (95% CI: 0.0–1.33) and OR: 0.21 (95% CI: 0.03–1.48), respectively.

Eleven placebo-controlled RCTs compared grade 3 and 4 cardiac AE for all four different categories of targeted agents in WD-NENs and MTC, investigated in our meta-analysis (Figure 4). Everolimus exhibited a pooled OR for serious cardiac AE of 3.28 (95% CI: 1.66–6.48) followed by TKIs with a pooled AE_OR of 1.51 (95% CI: 0.59–3.83). Within TKI analysis, sunitinib demonstrated the highest cardiac AE_OR in pancreatic NEN patients with OR figures as high as 7.17 (95% CI: 0.36–140.90), as compared to placebo. Surufatinib in pancreatic NENs, as well as vandetanib and cabozatinib in MTC do not appear to confer a risk for serious cardiac AE in the included RCTs (Figure 4).

With regards to biotherapy, SSAs demonstrated a potential prophylactic effect with respect to serious cardiac AE with an OR as low as 0.07 (95% CI: 0.0–1.33), which was comparable to that of telotristat etiprate (AE_OR: 0.21; 95% CI: 0.03–1.48; Figure 4).

Grade 3 and 4 cardiac AE in the 628 WD-NEN patients recieving everolimus in the intervention arm of the RADIANT trials included acute coronary syndrome (two patients), angina pectoris (two patients), cardiac arrest (two patients), cardiac failure (seven patients), congestive cardiac failure (six patients), cardio-respiratory arrest (two patients), left ventricular dysfunction/failure (two patients), right ventricular dysfunction (one patient), myocardial dysfunction (one patient), myocarditis (one patient), palpitations (one patient), tachycardia (one patient), pericardial efusion (two patients), tricuspid valve incompetence (one patient), mitral valve incompetence (one patient), pulmonary valve stenosis (one patient). 

In patients receiving TKIs, Grade 3 and 4 cardiac AE included atrial flutter (one patient), atrial fibrillation (two patients), cardiac failure (three patients), cardiopulmonary failure (one patient), left ventricular dysfunction (one patient), supraventricular tachycardia (one patient), right ventricular dysfunction (one patient), bradycardia (one patient), arrhythmia (one patient), and pericarditis (one patient). Finally, we did not observe any inter-study heterogeneity or publication bias within the different pooled analyses across different targeted NEN treatments (Figure 4 and Figure S3A–C).

### 2.7. Serious Vascular Toxicities’ Profile

**Figure 5:**
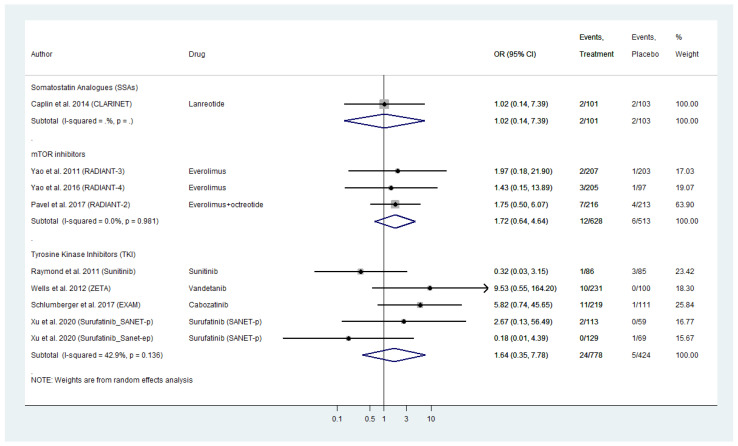
Targeted Agents’ Serious Vascular Toxicities profile per drug category in patients with Metastatic Well-Differentiated Neuroendocrine Neoplasms or Medullary Thyroid Cancer (Odds Ratio [OR] with 95% Confidence Intervals). Molecular targeted therapies were linked with higher risk for Serious Vascular Toxicities (Everolimus pooled OR: 1.72 [95% CI: 0.64–4.64]; Tyrosine kinase inhibitorsTKIs pooled OR of 1.64 [95% CI: 0.35–7.78]).

Nine placebo-controlled RCTs reported grade 3 and 4 vascular AE for WD-NENs treated with SSAs, everolimus or TKI and MTC treated with TKIs (Figure 5). Everolimus exhibited a pooled OR for serious vascular AE of 1.72 (95% CI: 0.64–4.64) followed by TKIs with a comparable pooled AE_OR of 1.64 (95% CI: 0.35–7.78). Within TKI analysis, vandetanib and cabozatinib, the two TKIs approved for MTC demonstrated the highest vascular AE_OR as high as 9.53 (95% CI: 0.55–164.60) for vandetanib and 5.82 (95% CI: 0.74–45.65) for cabozatinib, respectively when compared to placebo (Figure 5). With regards to biotherapy, SSAs in the CLARINET trial were not linked to a higher risk for serious vascular AE with an OR of 1.02 (95% CI: 0.14–7.39). The included placebo-controlled RCTs on telotristat etiprate did not report any serious vascular AEs.

The grade 3 and 4 vascular toxicities that were reported in the RADIANT trials included hypertension (two patients), hypotension (four patients), deep vein thrombosis (three patients), phlebitis, i.e., inflammation of the walls of a vein (one patient) and hematoma, i.e., a collection of blood outside of a blood vessel (one patient). Finally, the TKI trials reported the following serious vascular toxicities: hypertension (83 patients), hypertensive crisis (four patients), deep vein thrombosis (three patients) and hypotesion (one patient).

As in cardiac AEs’ pooled analyses, we did not observe any inter-study heterogeneity or publication bias (Figure 5 and Figure S4A–C).

### 2.8. Hypertension Secondary to Molecular Targeted Therapies

**Figure 6:**
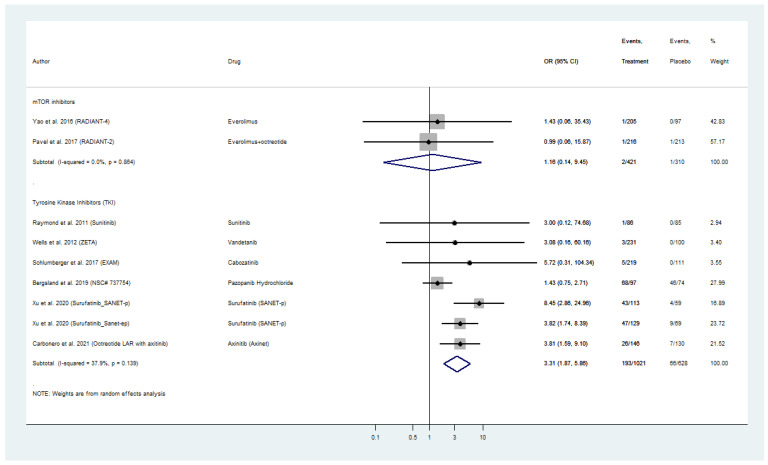
Targeted Agents’ Risk Assessment for new-onset or exacerbation of pre-existing Hypertension per drug category in patients with Metastatic Well-Differentiated Neuroendocrine Neoplasms or Medullary Thyroid Cancer (Odds Ratios with 95% Confidence Intervals). Tyrosine kinsae inhibitors exhibited a pooled OR for hypertension of 3.31 (95% CI: 1.87–5.86).

Nine placebo-controlled RCTs reported treatment-related hypertension, in particular new-onset or excarbation of pre-existing hyperension, in WD-NEN patients treated with everolimus or TKI and also MTC patients treated with TKIs (Figure 6). Everolimus was not linked to a significantly higher risk for treatment-related hypertension, as a pooled OR of 1.16 (95% CI: 0.14–9.45) was evident in RADIANT-2 and RADIANT-4 trials with only two cases of hypertension among 421 patients in the intervention arm. TKIs on the other hand exhibited a pooled OR for hypertension of 3.31 (95% CI: 1.87–5.86) with 193 patients with treatment-related hypertension among the 1021 patients in the intervention arm of these trials (Figure 6). Within TKI analysis, surufatinib in patients with pancreatic and extra-pancreatic NENs and cabozatinib in patients with MTC, demonstrated the highest ORs for treatment-related hypertension (SANET-p OR: 8.45; 95% CI: 2.86–24.96; SANET-ep OR: 3.82; 95% CI: 1.74–8.39; EXAM OR: 5.22; 95% CI: 0.31–104.34, respectively; Figure 6). The included placebo-controlled RCTs on biotherapy (SSAs and telotristat etiprate) did not report any treatment-related hypertension. Pooled analyses for hypertension were only available for everolimus and TKI, and did not reveal any inter-study heterogeneity or publication bias (Figure 6 and Figure S5A–C).

## 3. Discussion

In this systematic review and quantitative meta-analysis, we present all the available placebo-controlled randomized trials evaluating the safety profile of targeted therapies for metastatic WD-NEN and MTC with special focus on cardiovascular treatment-related toxicities. We identified sixteen RCTs that randomized 3408 patients with WD-NEN or MTC to biotherapy or MTTs. In general, the investigated targeted therapies exhibit a broad range of overall and grade 3 and 4 AE with regards to each drug’ distinct safety profile. In particular, analysis of grade 3 and 4 AE across both WD-NEN and MTC diagnoses, showed that there is evidence of a higher risk for serious toxicities in patients receiving the mTOR inhibitor everolimus and different TKIs and involved more commonly cardiovascular disorders for these agents, as compared to SSAs and telotristat etiprate. Furthermore, new onset or exacerbation of pre-existing hypertension was often encountered in patients that received TKI; with the highest risk being evident among patients that were administered the recently tested TKIs surufatinib and axitinib.

We applied the GRADE system to assess the risk of bias of the included placebo-controlled RCTs and were able to present high quality randomized evidence with a low risk of bias in most of the categories assessed (Table 2). However, inter-study heterogeneity was observed in the TKI subgroup meta-analysis for grade 3 and 4 as well as for all grade toxicities’ analyses. Complementary testing confirmed between study heterogeneity in our meta-analysis with respect to the aforementioned analyses. The studies that apparently contributed mostly to inter-study heterogeneity were the ones by Wells et al. on vandetanib for MTC (ZETA trial) and by Xu et al. on surufatinib far pancreatic NENs (SANET-p) [27,29]. Nevertheless, the studies included in our meta-analysis lacked the granularity to identify certain subsets of patients who may derive the most benefit from the administered treatments both in terms of therapeutic efficacy but also in terms of drug safety. For example, data on cardiovascular comorbidities, carcinoid heart disease, as well as detailed data on prior systemic therapies and potential additive toxicities were not available in the included studies. 

Targeted NEN agents have indeed been associated with a wide range of toxicities that may have an impact on the patients’ quality of life. Therefore, determining the timing and appropriate selection of the right agent to initiate the targeted treatment represents one of the most important future tasks, as for example the somatostatin receptor avidity per se is not sufficient to determine if a NEN patient is a good candidate for TPH inhibitors and MTT; and the currently available biomarkers, chromogranin A and 5-hydroxy indoloaceatic acid lack a predictive value with regards to treatment selection and monitoring response to biotherapy and MTTs [33,34]. In addition, the clinical efficacy of the investigated targeted therapies in WD-NEN and MTC have not been clearly associated with specific mutations, apart from cabozatinib for MTC in the EXAM trial, where a higher treatment effect was evident in patients with RET M918T mutant tumors [28]. Finally, it remains to be determined the exact sequencing of lines of treatments upon disease progression since there are many treatments not yet tested in trials with a head-to-head comparison or even placebo-controlled RCT of targeted agents, including immunotherapy, novel inhibitors of specific molecular targets, such as novel multikinases, MEK kinases and checkpoint immune factors.

In general, patients who could be candidates for biotherapy and MTTs should be counseled on the potential risks and benefits of this specificic therapy, as these drugs are linked with a disctinct safety profile, more commonly involving gastrointestinal AE and depression for biotherapy and a more diverse spectrum of AE also including cardiovascular AE for everolimus and TKIs. These AE have indeed a certain probability of negatively affecting patient compliance as well as cardiovascular health and overall quality of life, often necessitating dosage adaptations or therapy discontinuation. In our meta-analysis, NEN patients receiving the mTOR inhibitor everolimus or different TKIs exhibited a higher risk of developing serious (grade 3 and 4) or any other toxicities, lending further support to the notion that biotherapy appears to be a safer therapeutic approach with the least risk for treatment-related AE. Further analysis with a focus on cardiovascular health of NEN patients revealed that the placebo treatment has a similar or lower toxicity as compared to biotherapy (SSA and telotristat etiprate arms) pointing out the importance of recognizing the true causality of cardiovascular morbidity and the need to assess the potential prophylactic effect of biotherapy in cardiovascular health, for example in the context of carcinoid heart disease secondary to carcinoid syndrome in well-designed studies. Currently, the recommended monitoring for TKIs in context of renal cell carcinoma is mainly focused on blood pressure in agreement with the finding of our meta-analysis on higher risk for new onset or exacerbation of pre-existing hypertension following the administration of TKIs [35]. In addition, in patients receiving TKI therapy, cardiovascular events, including QTc prolongation, left ventricular HF, myocardial infarction (MI), hypertension, pulmonary hypertension, and stroke, were commonly reported by investigators [36]. For patients on sunitinib therapy in particular, baseline and periodic electrocardiograms are recommended [37].

However, besides therapy-induced cardiotoxicity, there are several other plausible interactions between cardiovascular health and NENs that still need to be determined as implicated above, with modulation of the immune system being an important player, but also the effect of serotonin, vasoactive substances and growth factors in cases of the carcinoid heart disease. For instance, the management of patients with poorly controlled hypertension or patients with a right heart failure due to fibrosis of right-sided valves may indeed be challenging and raise issues concerning treatment with targeted agents that could have an impact in patients’ already compromised cardiovascular health. Overall, despite their anti-tumor benefits, the use of MTTs and TKIs in particular has been hampered by potent cardiovascular toxicities, including hypertension. In addition, there is a paucity of real-world data on cardiovascular AEs caused by novel targeted agents in NENs and also a lack of studies on the pathophysiological mechanisms implicated in cardiovascular toxicity related to MTTs in this setting. As new cardiovascular AEs related to novel agents have emerged, clinicians managing NENs are now compelled to respond despite the lack of evidence regarding optimal management. Generally, routine monitoring of heart function and blood pressure during therapy with TKIs and identification of at risk patients before therapy seem to be the key steps in preventing cardiovascular events, regardless of the agent used. For patients on TKIs, baseline and periodic electrocardiograms are recommended. However, further studies are warranted to identify which of several targeted agents is at fault, acquire a complete understanding of the mechanisms of cardiovascular toxicity and provide follow-up guidelines specifically focusing on cardiovascular health with suggestions on modality and timing of toxicity prevention and management.

Our meta-analysis has some limitations. Some trials did not report on cardiovascular treatment-related AE and had an unclear risk of bias due to insufficiently reported data on random sequence generation, allocation concealment and blinding of outcome assessment. Due to the rather low number of studies included in the subgroup analysis of each drug category, the assessment of inter-study heterogeneity was limited. In addition, in the subset of MTC, tumors of more aggressive behavior might have been included. Furthermore, MTC patients were only eligible for treatment with two of the TKIs investigated in our study (vandetanib and cabozatinib); hence, the MTC trials have probably contributed to the heterogeneity of the included studies. Nevertheless, our study is also subject to confounding encountered in the original trials; thus, our results are only generalizable to patient groups that could be eligible for the original RCTs. Another limitation of our study, was the lack of detailed data on the number of new-onset and exacerbation of pre-existing hypertension, when assessing the safety profiles of TKIs for NEN patients. However, the strengths of our study was that it clearly provided its aim, as we applied a comprehensive search strategy, obtaining data also from unpublished placebo-controlled RCTs including all available randomized evidence according to Cochrane guidelines on the safety profile of targeted NEN therapies with a special focus on cardiovascular health, that could constitute a reference standard for clinicians in the future.

## 4. Materials and Methods

We followed the Cochrane Guidelines for Systematic Reviews and Meta-analyses of Interventions for the design and conduct of the present systematic review and quantitative meta-analysis and the Preferred Reporting Items for Systematic Reviews and Meta-Analyses (PRISMA) guidelines for reporting the study results [38,39].

### 4.1. Search Strategy and Study Selection

We aimed to identify all potentially eligible placebo-controlled RCTs comparing targeted therapies (biotherapy and MTTs) in metastatic WD-NEN or MTC. We developed a comprehensive search algorithm using MeSH terms and text words in the title or the abstract in combination with a systemic treatment, and a RCT study design filter. The search strategy for each database and the applied filters regarding treatment selection and study design are presented in Table S2. Only placebo-controlled RCTs on targeted therapies for NENs reporting data on safety were included. A study protocol for this systematic review was not registered in PROSPERO at the stage of inception owing to feasibility issues with respect to the specific nature of our study hypothesis and the pausity of potentially eligible NEN studies addressing cardiovascular toxicities. Importantly, a search was conducted to ensure that no similar systematic review had been previously published.

The PubMed, Embase, the Cochrane Central Register of Controlled Trials and the website of ClinicalTrials.gov were searched through until 1 December 2020. We did not apply any language or date restrictions. Key search terms included neuroendocrine neoplasms, medullary thyroid cancer, systemic therapy, and randomized controlled trial. We only included placebo-controlled RCTs comparing a targeted therapy with placebo reporting adverse-effect incidence with special focus in cardiovascular toxicities. Three of the authors (C.A., M.Y. and K.D.) worked in duplicates independently and screened all potentially eligible titles and abstracts and subsequently the full-text manuscripts to finalize eligibility. Disagreements were resolved by consensus between C.A., M.Y. and K.D. 

### 4.2. Outcomes and Data Extraction

The primary outcomes of this study were biotherapy’s and MTTs’ safety profiles. In particular, we assessed grade 3 and 4 (severe and life-threatening or disabling) AE, all grade AE, grade 3 and 4 cardiac AE, grade 3 and 4 vascular AE and treatment-related hypertension according to the National Cancer Institute Common Terminology Criteria for Adverse Events (CTCAE) v5.0 [14]. A CTCAE category is a broad classification of AEs based on anatomy and/or pathophysiology (for example, cardiac disorders; vascular disorders etc.) and within each category there are specific CTCAE terms that provide the standards for the description and exchange of safety information in oncology research. A list of serious and not serious treatment-related disorders as well as specific cardiac AEs and vascular AEs including hypertension were mainly obtained from clinicaltrials.gov and are provided in detail in Table S3.

Absolute values of AE were extracted and OR with 95% Confidence Intervals (CI) for PFS rates were calculated. Data on study-, patient- and tumor-characteristics, as well as industry sponsorship were also extracted. C.A., M.Y. and K.D. worked in duplicate and extracted all data independently. Discordances were resolved by consensus.

### 4.3. Risk of Bias and Quality of Evidence Assessment

We used the Grading of Recommendations Assessment, Development and Evaluation (GRADE) from the Cochrane Risk of Bias Tool to assess the risk of bias for the included RCTs [40]. We applied scores for standard domains: random sequence generation, allocation concealment, blinding of participants and personnel, blinding of outcome assessment, completeness of outcome data, for each domain. M.Y. and K.D. worked in duplicate and assessed the risk of bias and quality of evidence of the included RCTs. Disagreements were resolved by consensus. 

### 4.4. Statistical Analysis

The pooled estimates for the AE in patients with WD-NENS or MTC were assessed for the outcomes of interest and OR were calculated taking into account the correction of Haldane-Anscombe about 0 cells [41]. An OR is a relative measure of effect, which allows the comparison of the intervention group of a study relative to the placebo group. In particular, the OR represents the odds that an outcome will occur given a particular exposure (treatment) in the intervention group, compared to the odds of the outcome occurring in the absence of that exposure (administration of placebo) in the placebo group. The random variance component was estimated using the approach by Der Simonian and Laird [42]. To explore heterogeneity between the studies the I^2^ statistics were used. When I^2^ was> 0.50% the statistical heterogeneity was considered substantial [43]. Publication bias was assessed by the application of funnel plots and the Egger’s test to investigate the asymmetry among the study estimates [44]. All the analyses were performed using the STATA statistical package (version 13.1; StataCorp, College Station, TX, USA) 

## 5. Conclusions

Our systematic review and quantitative meta-analysis have implications for clinical practice and further research in the field of neuroendocrine tumors. It provides a comprehensive overview of the available randomized evidence on the safety profile of biotherapy (somatotostatin analogs and tryptophan hydroxylase inhibitors) and that of molecular targeted therapies (mTOR and tyrosine kinase inhibitors) with a special focus on cardiovascular treatment-related toxicities Somatotostatin analogs and tryptophan hydroxylase inhibitors appear to be safer as compared to mTOR and tyrosine kinase inhibitors with regards to their overall toxicity profile, and cardiovascular toxicities in particular. In addition, new onset or exacerbation of pre-existing hypertension was often encountered in patients who received tyrosine kinase inhibitors. Apart from evidence on the efficacy of biotherapy and MTTs, data on treatment-related toxicities and the distinct safety profile of each agent could promote a patient-tailored approach guiding clinicians’ treatment decisions, but also patient surveillance with early recognition and prompt management of treatment-related toxicities when they appear. Finally, our results highlight the need for further research in assessing long-term real world-data on cardiovascular health as well as effects on quality of life of patients receiving different targeted NEN therapies in order to achieve a balance between antitumor activity and toxicities. This is of course most probably accomplished when NEN patients receiving multimodal treatments are managed in dedicated centers with treatment decisions being taken in the context of a multidisciplinary tumor board.

## Figures and Tables

**Table 1:** Characteristics of placebo-controlled randomized controlled trials included in the meta-analysis.

Study	Drug	Origin	Type of Treatment	Median Treatment Duration	Median Follow-Up [Months]	Complete Follow-Up [%]	Sample Size Calculation	N Participants Randomized	Included in Enets, ATA/ETA Guidelines	Industry Sponsorship
SSAs										
Caplin et al., 2014 [17] (CLARINET)	Lanreotide	14 countries	Lanreotide 120 mg/28 d Placebo	24.0 15.0	n.a n.a.	100	Yes	101 103	Yes	Yes
Rinke et al., 2009 [18] (PROMID).	Octreotide LAR	Germany	Octreotide LAR 30 mg/28 d Placebo	n.d n.d.	n.a. n.a.	99	Yes	42 43	Yes	Yes
Vinik et al., 2016 [19] (ELECT).	Lanreotide	11 countries	Lanreotide 120 mg/4weeks Placebo	4.6 3.7	n.a. n.a.	99	Yes	86 85	Yes	Yes
TPH Inhibitors										
Kulke et al., 2017 [20] (TELESTAR).	Telotristat etiprate	12 countries	Telotristat ethyl 250 mg or 500 mg three times per day or placebo three times per day	12.0 12.0	n.a. n.a.	100	Yes	45 45 45	No	Yes
Pavel et al., 2018 [21] (TELECAST).	Telotristat ethyl	11 counties	Telotristat ethyl 250 mg tid or 500 mg tid or placebo	12.0 12.0	36	89	Yes	26 25 25	No	Yes
Kulke et al., 2014 [22]	Telotristat etiprate	USA	telotristat etiprate 150 mg or 250 mg or 350 mg or 500 mg tid or placebo	4.0 4.0 4.0 4.0 4.0	n.a. n.a.	95	Yes	5 3 3 3 9	No	Yes
mTOR Inhibitors										
Pavel et al., 2017 [23] (RADIANT-2).	Everolimus + octreotide	16 countries	Everolimus 10 mg/d + octreotide LAR 30 mg/28 d Placebo + octreotide LAR 30 mg/28 d	9.3 9.2	n.a. n.a.	100	Yes	216 213	Yes	Yes
Yao et al., 2011 [24] (RADIANT-3).	Everolimus	18 countries	Everolimus 10 mg/d Placebo	8.8 3.7	17 17	62	Yes	207 203	Yes	Yes
Yao et al., 2016 [25] (RADIANT-4).	Everolimus	25 countries	Everolimus 10 mg/d Placebo	9.3 4.5	21 21	Above 80	Yes	205 97	Yes	Yes
TKI										
Raymond et al., 2011 [26]	Sunitinib	11 countries	Sunitinib 37.5 mg/d Placebo	4.6 3.7	n.a. n.a.	99	Yes	86 85	Yes	Yes
Wells et al., 2012 [27] (ZETA)	Vandetanib	3 countries	Vandetanib 300 mg vs. placebo	n.a. n.a.	24	100	Yes	330	Yes	Yes
Schlumberger et al., 2017 [28] (EXAM).	Cabozatinib	Germany	Cabozantinib vs. placebo	n.a. n.a.	n.a. n.a.	99.7	Yes	330	No	Yes
Xu et al., 2020 [29] (SANET-p)	Surufatinib	China	Surufatinib 300 mg vs. placebo	7.6 4.1	19.3 11.1	n.a	Yes	172	No	Yes
Carbonero et al., 2021 [30] (AXINET)	Axinitib	NA	Axitinib 5 mg BID + Sandostatin LAR 30 mg/28 days	n.a. n.a.	n.a. n.a.	n.a.	Yes	126 130	No	Yes
Bergland et al. [31] 2019	Pazopanib Hydrochloride		Pazopanib 800 mg PO QD on days 1–28 vs. placebo	60 60	60 60	100	Yes	97 74	No	Yes
Xu et al., 2020 [32] (SANET-ep).	Surufatinib	China	Surufatinib 300 mg vs. placebo	7.1 4.8	13.8 16.6	100	Yes	88 53	No	Yes

Abbreviations. ATA: American Thyroid Association; ENETS: European Neuroendocrine Tumor Society; ETA: European Thyroid Association; mTOR: Mechanistic target of rapamycin; n.a.: Non available; SSAs: Somatostatin analogs; TKI: Tyrosine kinase inhibitor; TPH: Tryptophan hydroxylase.

**Table 2:** Risk of bias summary: Authors’ judgments about each risk of bias item for each included study following the Grading of Recommendations Assessment, Development and Evaluation (GRADE) Approach.

Study	Random Sequence Generation	Allocation Concealment	Blinding of Participants and Personnel	Blinding of Outcome Assessment	Incomplete Outcome Data	Selective Reporting	Other Bias
Caplin et al., 2014 [17] (CLARINET).	(-)	(-)	(-)	(-)	(-)	(-)	?
Rinke et al., 2009 [18] (PROMID)	(-)	(-)	(-)	(-)	(-)	(-)	?
Vinik et al., 2016 [19] (ELECT).	(-)	(-)	(-)	?	(-)	(-)	(-)
Kulke et al., 2017 [20] (TELESTAR).	?	?	?	?	(-)	(-)	(-)
Pavel et al., 2018 [21] (TELECAST).	(-)	(-)	(+)	(+)	(-)	(-)	(-)
Kulke et al., 2014 [22]	?	?	?	?	(-)	(-)	(-)
Pavel et al., 2017 [23] (RADIANT-2).	?	(-)	(-)	(-)	(-)	(-)	?
Yao et al., 2011 (RADIANT-3) [24].	(-)	(-)	(-)	(-)	(-)	(-)	?
Yao et al., 2016 (RADIANT-4) [25].	(-)	(-)	?	?	(-)	(-)	?
Raymond et al., 2011 [26]	?	?	(-)	(-)	?	(-)	?
Wells et al., 2012 [27] (ZETA).	?	?	(-)	(-)	(-)	(-)	?
Schlumberger et al. [28] 2017 (EXAM).	?	?	(-)	(-)	(-)	(-)	?
Xu et al., 2020 [29] (SANET-p).	(+)	(+)	(+)	(+)	(+)	(-)	?
Carbonero et al., 2021 [30] (AXINET)	(+)	?	(+)	(+)	?	(-)	(-)
Bergsland et al. [31] 2019	(+)	?	(+)	?	(-)	(-)	?
Xu et al., 2020 [32] (SANET-ep).	(+)	(+)	(+)	(+)	(+)	(-)	(-)

Each domain was judged as ‘low risk of bias’ (-), ‘high risk of bias’ (+), or ‘unclear risk of bias’ (?) in each study according to the Cochrane Handbook for Systematic Reviews of Interventions 1.

## Data Availability

Individual patient data from the original studies included in the present meta-analysis is not available and data sharing at this level is not applicable for a systematic review.

## Supplementary Materials

The following are available online at https://www.mdpi.com/article/10.3390/cancers13092159/s1, Figure S1A: Funnel plot for studies included in the pooled analysis for Grade 3 and 4 Adverse Effects; S1B: Galbraith’s plot for studies included in this analysis; S1C: Egger’s plot for studies included in this analysis, Figure S2A: Funnel plot for studies included in the pooled analysis for all Grade Adverse Effects; S2B: Galbraith’s plot for studies included in this analysis; S2C: Egger’s plot for studies included in this analysis, Figure S3A: Funnel plot for studies included in the pooled analysis for Grade 3 and 4 Cardiac Adverse Effects; S3B: Galbraith’s plot for studies included in this analysis; S3C: Egger’s plot for studies included in this analysis, Figure S4A: Funnel plot for studies included in the pooled analysis for Grade 3 and 4 Vascular Adverse Effects; S4B: Galbraith’s plot for studies included in this analysis; S4C: Egger’s plot for studies included in this analysis, Figure S5A: Funnel plot for studies included in the pooled analysis for treatment-related Hypertension; S5B: Galbraith’s plot for studies included in this analysis; S5C: Egger’s plot for studies included in this analysis, Table S1: Patient and tumor characteristics across the included studies. Table S2: Systematic literature search strategy, Table S3: A. List of serious and not-serious disorders B. Specific cardiac adverse effects and C. Specific vascular adverse effects including hypertension assessed in the present study in order to categorize toxicities into different safety profiles (serious toxicities, all grade toxicities, serious cardiac toxicities, vascular toxicities and hypertension) according to the National Cancer Institute Common Terminology Criteria for Adverse Events v5.0.

## References

[1] Dasari A., Shen C., Halperin D., Zhao B., Zhou S., Xu Y., Shih T., Yao J.C. (2017). Trends in the Incidence, Prevalence, and Survival Outcomes in Patients with Neuroendocrine Tumors in the United States. JAMA Oncol..

[2] Roman S., Lin R., Sosa J.A. (2006). Prognosis of medullary thyroid carcinoma: Demographic, clinical, and pathologic predictors of survival in 1252 cases. Cancer.

[3] Alexandraki K.I., Kaltsas G. (2012). Gastroenteropancreatic neuroendocrine tumors: New insights in the diagnosis and therapy. Endocrine.

[4] Pavel M., Valle J.W., Eriksson B., Rinke A., Caplin M., Chen J., Costa F., Falkerby J., Fazio N., Gorbounova V. (2017). ENETS Consensus Guidelines for the Standards of Care in Neuroendocrine Neoplasms: Systemic Therapy—Biotherapy and Novel Targeted Agents. Neuroendocrinology.

[5] Kaderli R.M., Spanjol M., Kollar A., Butikofer L., Gloy V., Dumont R.A., Seiler C.A., Christ E.R., Radojewski P., Briel M. (2019). Therapeutic Options for Neuroendocrine Tumors: A Systematic Review and Network Meta-analysis. JAMA Oncol..

[6] Oleinikov K., Korach A., Planer D., Gilon D., Grozinsky-Glasberg S. (2021). Update in carcinoid heart disease—The heart of the matter. Rev. Endocr. Metab. Disord..

[7] Liu M., Armeni E., Navalkissoor S., Davar J., Sullivan L., Leigh C., O’Mahony L.F., Hayes A., Mandair D., Chen J. (2020). Cardiac Metastases in Patients with Neuroendocrine Tumours: Clinical Features, Therapy Outcomes, and Prognostic Implications. Neuroendocrinology.

[8] Alexandraki K.I., Daskalakis K., Tsoli M., Grossman A.B., Kaltsas G.A. (2020). Endocrinological Toxicity Secondary to Treatment of Gastroenteropancreatic Neuroendocrine Neoplasms (GEP-NENs). Trends Endocrinol. Metab..

[9] Heidarpour M., Shafie D., Aminorroaya A., Sarrafzadegan N., Farajzadegan Z., Nouri R., Najimi A., Dimopolou C., Stalla G. (2019). Effects of somatostatin analog treatment on cardiovascular parameters in patients with acromegaly: A systematic review. J. Res. Med. Sci..

[10] Denney W.D., Kemp W.E., Anthony L.B., Oates J.A., Byrd B.F. (1998). Echocardiographic and biochemical evaluation of the development and progression of carcinoid heart disease. J. Am. Coll. Cardiol..

[11] Cheng H., Force T. (2010). Molecular mechanisms of cardiovascular toxicity of targeted cancer therapeutics. Circ. Res..

[12] Liu K.L., Chen J.S., Chen S.C., Chu P.H. (2013). Cardiovascular Toxicity of Molecular Targeted Therapy in Cancer Patients: A Double-Edged Sword. Acta Cardiol. Sin..

[13] Hasinoff B.B. (2010). The cardiotoxicity and myocyte damage caused by small molecule anticancer tyrosine kinase inhibitors is correlated with lack of target specificity. Toxicol. Appl. Pharmacol..

[14] Common Terminology Criteria for Adverse Events v5.0 (CTCAE). https://ctep.cancer.gov/protocoldevelopment/electronic_applications/ctc.htm.

[15] Wells S.A., Asa S.L., Dralle H., Elisei R., Evans D.B., Gagel R.F., Lee N., Machens A., Moley J.F., Pacini F. (2015). Revised American Thyroid Association guidelines for the management of medullary thyroid carcinoma. Thyroid.

[16] Schlumberger M., Bastholt L., Dralle H., Jarzab B., Pacini F., Smit J.W., European Thyroid Association Task F. (2012). 2012 European thyroid association guidelines for metastatic medullary thyroid cancer. Eur. Thyroid. J..

[17] Caplin M.E., Pavel M., Cwikla J.B., Phan A.T., Raderer M., Sedlackova E., Cadiot G., Wolin E.M., Capdevila J., Wall L. (2014). Lanreotide in metastatic enteropancreatic neuroendocrine tumors. N. Eng. J. Med..

[18] Rinke A., Muller H.-H., Schade-Brittinger C., Klose K.-J., Barth P., Wied M., Mayer C., Aminossadati B., Pape U.-F., Blaker M. (2009). Placebo-controlled, double-blind, prospective, randomized study on the effect of octreotide LAR in the control of tumor growth in patients with metastatic neuroendocrine midgut tumors: A report from the PROMID Study Group. J. Clin. Oncol. Off. J. Am. Soc. Clin. Oncol..

[19] Vinik A.I., Wolin E.M., Liyanage N., Gomez-Panzani E., Fisher G.A. (2016). Evaluation of lanreotide depot/autogel efficacy and safety as a carcinoid syndrome treatment (elect): A randomized, double-blind, placebo-controlled trial. Endocr. Prac. Off. J. Am. Coll. Endocrinol. Am. Assoc. Clin. Endocrinol..

[20] Kulke M.H., Horsch D., Caplin M.E., Anthony L.B., Bergsland E., Oberg K., Welin S., Warner R.R.P., Lombard-Bohas C., Kunz P.L. (2017). Telotristat Ethyl, a Tryptophan Hydroxylase Inhibitor for the Treatment of Carcinoid Syndrome. J. Clin. Oncol. Off. J. Am. Soc. Clin. Oncol..

[21] Pavel M., Gross D.J., Benavent M., Perros P., Srirajaskanthan R., Warner R.R.P., Kulke M.H., Anthony L.B., Kunz P.L., Horsch D. (2018). Telotristat ethyl in carcinoid syndrome: Safety and efficacy in the TELECAST phase 3 trial. Endocr. Relat. Cancer.

[22] Kulke M.H., O’Dorisio T., Phan A., Bergsland E., Law L., Banks P., Freiman J., Frazier K., Jackson J., Yao J.C. (2014). Telotristat etiprate, a novel serotonin synthesis inhibitor, in patients with carcinoid syndrome and diarrhea not adequately controlled by octreotide. Endocr. Relat. Cancer.

[23] Pavel M.E., Baudin E., Oberg K.E., Hainsworth J.D., Voi M., Rouyrre N., Peeters M., Gross D.J., Yao J.C. (2017). Efficacy of everolimus plus octreotide LAR in patients with advanced neuroendocrine tumor and carcinoid syndrome: Final overall survival from the randomized, placebo-controlled phase 3 RADIANT-2 study. Ann. Oncol. Off. J. Eur. Soc. Med. Oncol..

[24] Yao J.C., Shah M.H., Ito T., Bohas C.L., Wolin E.M., Van Cutsem E., Hobday T.J., Okusaka T., Capdevila J., de Vries E.G.E. (2011). Everolimus for advanced pancreatic neuroendocrine tumors. N. Eng. J. Med..

[25] Yao J.C., Fazio N., Singh S., Buzzoni R., Carnaghi C., Wolin E., Tomasek J., Raderer M., Lahner H., Voi M. (2016). Everolimus for the treatment of advanced, non-functional neuroendocrine tumours of the lung or gastrointestinal tract (RADIANT-4): A randomised, placebo-controlled, phase 3 study. Lancet.

[26] Raymond E., Dahan L., Raoul J.-L., Bang Y.-J., Borbath I., Lombard-Bohas C., Valle J., Metrakos P., Smith D., Vinik A. (2011). Sunitinib malate for the treatment of pancreatic neuroendocrine tumors. N. Eng. J. Med..

[27] Wells S.A., Robinson B.G., Gagel R.F., Dralle H., Fagin J.A., Santoro M., Baudin E., Elisei R., Jarzab B., Vasselli J.R. (2012). Vandetanib in patients with locally advanced or metastatic medullary thyroid cancer: A randomized, double-blind phase III trial. J. Clin. Oncol. Off. J. Am. Soc. Clin. Oncol..

[28] Schlumberger M., Elisei R., Müller S., Schöffski P., Brose M., Shah M., Licitra L., Krajewska J., Kreissl M.C., Niederle B. (2017). Overall survival analysis of EXAM, a phase III trial of cabozantinib in patients with radiographically progressive medullary thyroid carcinoma. Ann. Oncol. Off. J. Eur. Soc. Med. Oncol..

[29] Xu J., Shen L., Bai C., Wang W., Li J., Yu X., Li Z., Li E., Yuan X., Chi Y. (2020). Surufatinib in advanced pancreatic neuroendocrine tumours (SANET-p): A randomised, double-blind, placebo-controlled, phase 3 study. Lancet Oncol..

[30] Rocio Garcia-Carbonero M.B., Fonseca P.J., Castellano D., Alonso T., Teule A., Custodio A., Tafuto S., Munoa A.L., Spada F., López-López C. (2021). A phase II/III randomized double-blind study of octreotide acetate LAR with axitinib versus octreotide acetate LAR with placebo in patients with advanced G1-G2 NETs of non-pancreatic origin (AXINET trial-GETNE-1107). J. Clin. Oncol..

[31] Bergsland E.K., Mahoney M.R., Asmis T.R., Hall N., Kumthekar P., Maitland M.L., Niedzwiecki D., Nixon A.B., O’Reilly E.M., Schwartz L.H. (2019). Meyerhardt. Prospective randomized phase II trial of pazopanib versus placebo in patients with progressive carcinoid tumors (CARC) (Alliance A021202). Am. Soc. Clin. Oncol..

[32] Xu J., Shen L., Zhou Z., Li J., Bai C., Chi Y., Li Z., Xu N., Li E., Liu T. (2020). Surufatinib in advanced extrapancreatic neuroendocrine tumours (SANET-ep): A randomised, double-blind, placebo-controlled, phase 3 study. Lancet Oncol..

[33] Dam G., Gronbaek H., Sorbye H., Thiis Evensen E., Paulsson B., Sundin A., Jensen C., Ebbesen D., Knigge U., Tiensuu Janson E. (2020). Prospective Study of Chromogranin A as a Predictor of Progression in Patients with Pancreatic, Small-Intestinal, and Unknown Primary Neuroendocrine Tumors. Neuroendocrinology.

[34] Wedin M., Mehta S., Angeras-Kraftling J., Wallin G., Daskalakis K. (2021). The Role of Serum 5-HIAA as a Predictor of Progression and an Alternative to 24-h Urine 5-HIAA in Well-Differentiated Neuroendocrine Neoplasms. Biology.

[35] Hall P.S., Harshman L.C., Srinivas S., Witteles R.M. (2013). The frequency and severity of cardiovascular toxicity from targeted therapy in advanced renal cell carcinoma patients. JACC Heart Fail..

[36] Schmidinger M., Zielinski C.C., Vogl U.M., Bojic A., Bojic M., Schukro C., Ruhsam M., Hejna M., Schmidinger H. (2008). Cardiac toxicity of sunitinib and sorafenib in patients with metastatic renal cell carcinoma. J. Clin. Oncol..

[37] Richards C.J., Je Y., Schutz F.A., Heng D.Y., Dallabrida S.M., Moslehi J.J., Choueiri T.K. (2011). Incidence and risk of congestive heart failure in patients with renal and nonrenal cell carcinoma treated with sunitinib. J. Clin. Oncol..

[38] Cumpston M., Li T., Page M.J., Chandler J., Welch V.A., Higgins J.P., Thomas J. (2019). Updated guidance for trusted systematic reviews: A new edition of the Cochrane Handbook for Systematic Reviews of Interventions. Cochrane Database Syst. Rev..

[39] Moher D., Liberati A., Tetzlaff J., Altman D.G., Group P. (2009). Preferred reporting items for systematic reviews and meta-analyses: The PRISMA statement. BMJ.

[40] Guyatt G.H., Oxman A.D., Vist G.E., Kunz R., Falck-Ytter Y., Alonso-Coello P., Schunemann H.J., Group G.W. (2008). GRADE: An emerging consensus on rating quality of evidence and strength of recommendations. BMJ.

[41] Lawson R. (2004). Small sample confidence intervals for the odds ratio. Commun. Stat. Simul. Comput..

[42] DerSimonian R., Laird N. (1986). Meta-analysis in clinical trials. Control. Clin. Trials.

[43] Higgins J.P., Thompson S.G. (2002). Quantifying heterogeneity in a meta-analysis. Stat. Med..

[44] Sterne J.A., Sutton A.J., Ioannidis J.P., Terrin N., Jones D.R., Lau J., Carpenter J., Rucker G., Harbord R.M., Schmid C.H. (2011). Recommendations for examining and interpreting funnel plot asymmetry in meta-analyses of randomised controlled trials. BMJ.

