# Supplementary material for: Cardiovascular Toxicities Secondary to Biotherapy and Molecular Targeted Therapies in Neuroendocrine Neoplasms: A Systematic Review and Meta-Analysis of Randomized Placebo-Controlled Trials
Source: Cancers (Basel). 2021 Apr 30;13(9):2159. doi: 10.3390/cancers13092159 (PMC8124890; doi:10.3390/cancers13092159)
Supplement: Supplementary file 1 [file cancers-13-02159-s001.zip › cancers-1172970-SI.pdf]

cancers

MDPI

Supplementary Materials: Cardiovascular Toxicities Secondary to Biotherapy and Molecular Targeted Therapies in Neuroendocrine Neoplasms: A Systematic Review and Meta-Analysis of Randomized Placebo-Controlled Trials

Charalampos Aktypis, Maria-Eleni Spei, Maria Yavropoulou, Göran Wallin, Anna Koumarianou, Gregory Kaltsas, Eva Kassi and Kosmas Daskalakis

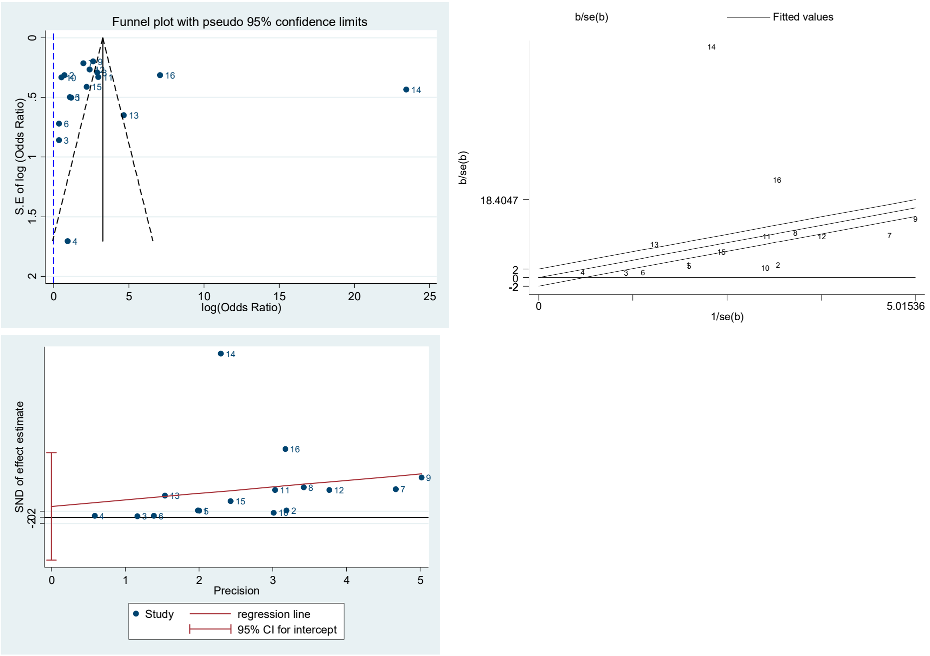

Figure S1A: Funnel plot for studies included in the pooled analysis for Grade 3 and 4 Adverse Effects; S1B: Galbraith's plot for studies included in this analysis; S1C: Egger's plot for studies included in this analysis

study	author	drug	drugtype
1	Rinke et al. 2009 (PROMID)	Octreotide LAR	Somatostatin Analogues (SSAs)
2	Caplin et al. 2014 (CLARINET)	Lanreotide	Somatostatin Analogues (SSAs)
3	Vinik et al. 2016 (ELECT)	Lanreotide	Somatostatin Analogues (SSAs)
4	Kulke et al. 2014 (Telotristat etipra	Telotristat etiprate	Tryptophan Hydrohylase (TPH) inhibitor
5	Kulke et al. 2017 (TELESTAR)	Telotristat etiprate	Tryptophan Hydrohylase (TPH) inhibitor
6	Pavel et al. 2018 (telotristat ethyl_	Telotristat ethyl (TELECAS	Tryptophan Hydrohylase (TPH) inhibitor
7	Yao et al. 2011 (RADIANT-3)	Everolimus	mTOR inhibitors
8	Yao et al. 2016 (RADIANT-4)	Everolimus	mTOR inhibitors
9	Pavel et al. 2017 (RADIANT-2)	Everolimus+octreotide	mTOR inhibitors
10	Raymond et al. 2011 (Sunitinib)	Sunitinib	Tyrosine Kinase Inhibitors (TKI)
11	Wells et al. 2012 (ZETA)	Vandetanib	Tyrosine Kinase Inhibitors (TKI)
12	Schlumberger et al. 2017 (EXAM)	Cabozatinib	Tyrosine Kinase Inhibitors (TKI)
13	Bergsland et al. 2019 (NSC# 73775	Pazopanib Hydrochloride	Tyrosine Kinase Inhibitors (TKI)
14	Xu et al. 2020 (Surufatinib_SANET-	Surufatinib (SANET-p)	Tyrosine Kinase Inhibitors (TKI)
15	Xu et al. 2020 (Surufatinib_Sanet-e	Surufatinib (SANET-p)	Tyrosine Kinase Inhibitors (TKI)
16	Carbonero et al. 2021 (Octreotide	Axinitib (Axinet)	Tyrosine Kinase Inhibitors (TKI)

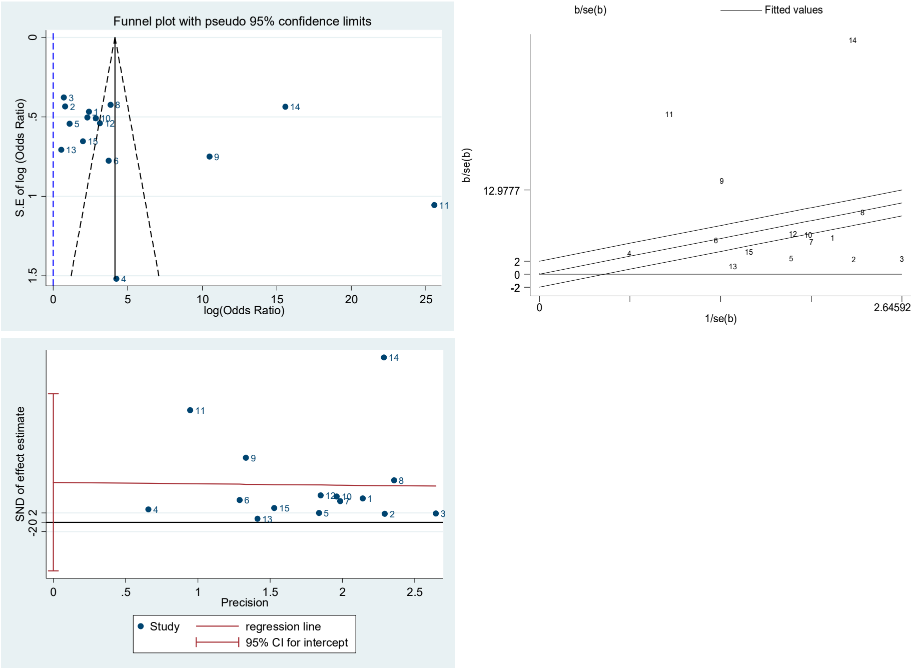

Figure S2A: Funnel plot for studies included in the pooled analysis for all Grade Adverse Effects; S2B: Galbraith's plot for studies included in this analysis; S2C: Egger's plot for studies included in this analysis

study	author	drug	drugtype
1	Rinke et al. 2009 (PROMID)	Octreotide LAR	Somatostatin Analogues (SSAs)
2	Caplin et al. 2014 (CLARINET)	Lanreotide	Somatostatin Analogues (SSAs)
3	Vinik et al. 2016 (ELECT)	Lanreotide	Somatostatin Analogues (SSAs)
4	Kulke et al. 2014 (Telotristat etipra	Telotristat etiprate	Tryptophan Hydrohylase (TPH) inhibitors
5	Kulke et al. 2017 (TELESTAR)	Telotristat etiprate	Tryptophan Hydrohylase (TPH) inhibitors
6	Pavel et al. 2018 (telotristat ethyl_	Telotristat ethyl (TELECAST)	Tryptophan Hydrohylase (TPH) inhibitors
7	Yao et al. 2011 (RADIANT-3)	Everolimus	mTOR inhibitors
8	Yao et al. 2016 (RADIANT-4)	Everolimus	mTOR inhibitors
9	Pavel et al. 2017 (RADIANT-2)	Everolimus+octreotide	mTOR inhibitors
10	Raymond et al. 2011 (Sunitinib)	Sunitinib	Tyrosine Kinase Inhibitors (TKI)
11	Wells et al. 2012 (ZETA)	Vandetanib	Tyrosine Kinase Inhibitors (TKI)
12	Schlumberger et al. 2017 (EXAM)	Cabozatinib	Tyrosine Kinase Inhibitors (TKI)
13	Bergsland et al. 2019 (NSC# 73775	Pazopanib Hydrochloride	Tyrosine Kinase Inhibitors (TKI)
14	Xu et al. 2020 (Surufatinib_Sanet-e	Surufatinib (SANET-p)	Tyrosine Kinase Inhibitors (TKI)
15	Xu et al. 2020 (Surufatinib_SANET-	Surufatinib (SANET-p)	Tyrosine Kinase Inhibitors (TKI)

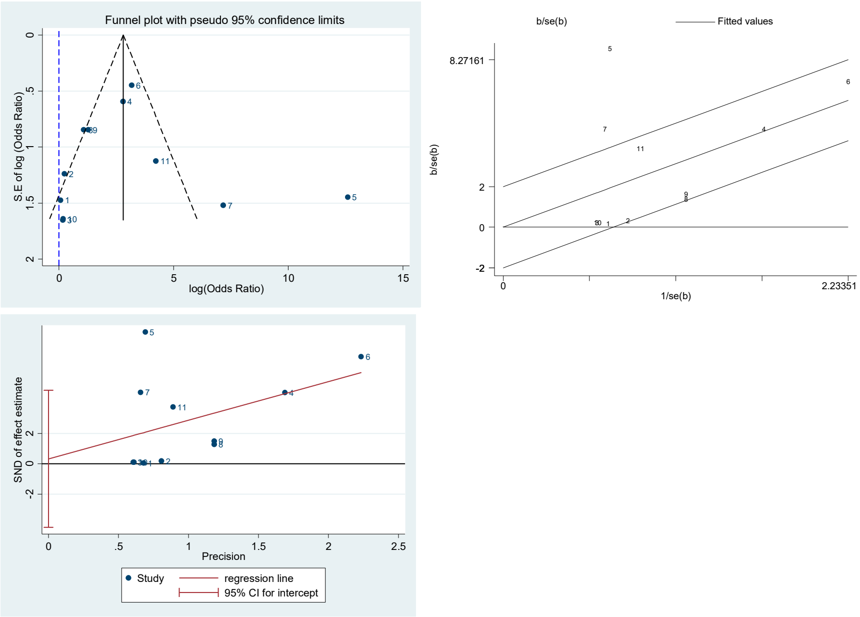

Figure S3A: Funnel plot for studies included in the pooled analysis for Grade 3 and 4 Cardiac Adverse Effects; S3B: Galbraith's plot for studies included in this analysis; S3C: Egger's plot for studies included in this analysis

study	author	drug	drugtype
1	Caplin et al. 2014 (CLARINET)	Lanreotid	Somatostatin Analogues (SSAs)
2	Kulke et al. 2017 (TELESTAR)	Telotristat	Tryptophan Hydrohylase (TPH)
3	Pavel et al. 2018 (telotristat ethyl_	Telotristat	Tryptophan Hydrohylase (TPH)
4	Yao et al. 2011 (RADIANT-3)	Everolimu	mTOR inhibitors
5	Yao et al. 2016 (RADIANT-4)	Everolimu	mTOR inhibitors
6	Pavel et al. 2017 (RADIANT-2)	Everolimu	mTOR inhibitors
7	Raymond et al. 2011 (Sunitinib)	Sunitinib	Tyrosine Kinase Inhibitors (TKI)
8	Wells et al. 2012 (ZETA)	Vandetani	Tyrosine Kinase Inhibitors (TKI)
9	Schlumberger et al. 2017 (EXAM)	Cabozatin	Tyrosine Kinase Inhibitors (TKI)
10	Xu et al. 2020 (Surufatinib_Sanet-e	Surufatini	Tyrosine Kinase Inhibitors (TKI)
11	Carbonero et al. 2021 (Octreotide	Axinitib (A	Tyrosine Kinase Inhibitors (TKI)

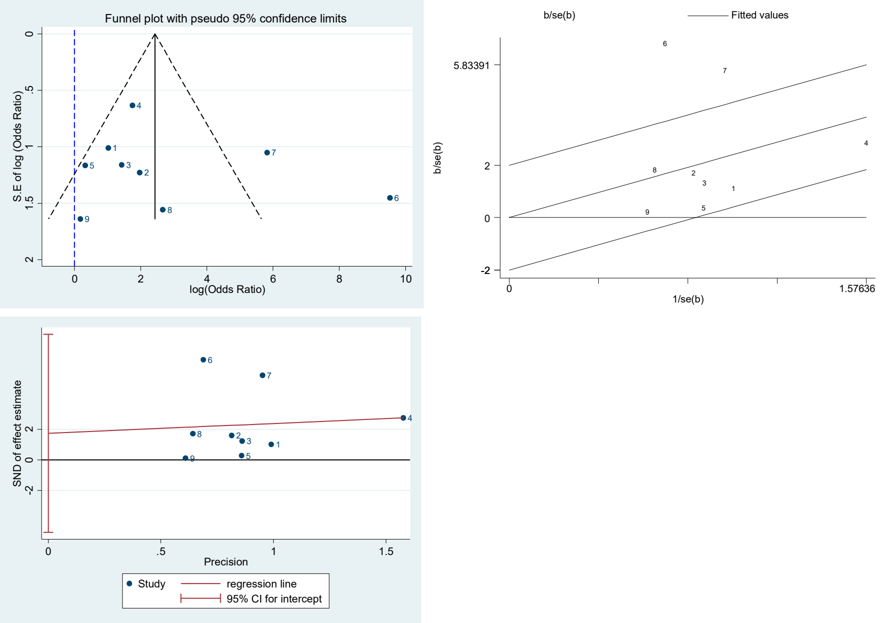

Figure S4A: Funnel plot for studies included in the pooled analysis for Grade 3 and 4 Vascular Adverse Effects; S4B: Galbraith's plot for studies included in this analysis; S4C: Egger's plot for studies included in this analysis

study	author	drug	drugtype
1	Caplin et al. 2014 (CLARINET)	Lanreotid	Somatostatin Analogues (S
2	Yao et al. 2011 (RADIANT-3)	Everolimu	mTOR inhibitors
3	Yao et al. 2016 (RADIANT-4)	Everolimu	mTOR inhibitors
4	Pavel et al. 2017 (RADIANT-2)	Everolimu	mTOR inhibitors
5	Raymond et al. 2011 (Sunitinib)	Sunitinib	Tyrosine Kinase Inhibitors
6	Wells et al. 2012 (ZETA)	Vandetani	Tyrosine Kinase Inhibitors
7	Schlumberger et al. 2017 (EXAM)	Cabozatin	Tyrosine Kinase Inhibitors
8	Xu et al. 2020 (Surufatinib_SANET-	Surufatini	Tyrosine Kinase Inhibitors
9	Xu et al. 2020 (Surufatinib_Sanet-e	Surufatini	Tyrosine Kinase Inhibitors

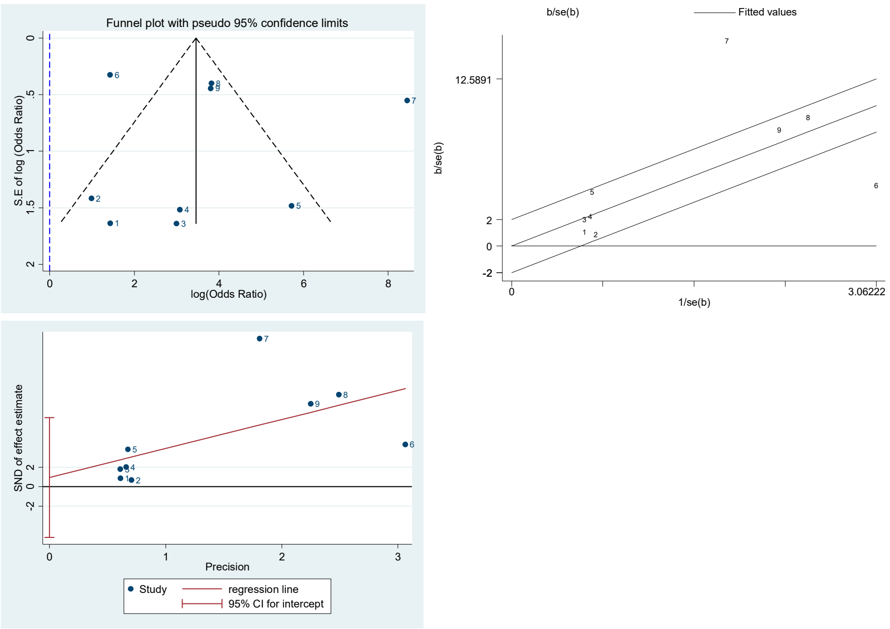

Figure S5A: Funnel plot for studies included in the pooled analysis for treatment-related Hypertension; S5B: Galbraith's plot for studies included in this analysis; S5C: Egger's plot for studies included in this analysis

study	author	drug	drugtype
1	Yao et al. 2016 (RADIANT-4)	Everolimu	mTOR inhibitors
2	Pavel et al. 2017 (RADIANT-2)	Everolimu	mTOR inhibitors
3	Raymond et al. 2011 (Sunitinib	Sunitinib	Tyrosine Kinase Inhibitors (TKI)
4	Wells et al. 2012 (ZETA)	Vandetani	Tyrosine Kinase Inhibitors (TKI)
5	Schlumberger et al. 2017 (EXAN	Cabozatin	Tyrosine Kinase Inhibitors (TKI)
6	Bergsland et al. 2019 (NSC# 73	Pazopanib	Tyrosine Kinase Inhibitors (TKI)
7	Xu et al. 2020 (Surufatinib_SAN	Surufatini	Tyrosine Kinase Inhibitors (TKI)
8	Xu et al. 2020 (Surufatinib_San	Surufatini	Tyrosine Kinase Inhibitors (TKI)
9	Carbonero et al. 2021 (Octreoti	Axinitib (A	Tyrosine Kinase Inhibitors (TKI)

Table 1. Patient and tumor characteristics.

Study	Drug	Primary tumor site	Grading (% of patients included)	Functional tumors [%]	Females [%]	Median or mean age [years]
	**SSAs**					
Caplin et al. 2014 (CLARINET).	Lanreotide	GI , Lung	G1 (68), G2 (32)<br>G1 (70), G2 (28), unknown (2)	2<br>2	48<br>48	Mean: 63<br>Mean: 62
Rinke et al. 2009 (PROMID).	Octreotide LAR	GI	G1 (98), G2 (2) G1 (93), G2 (7)	41<br>37	52<br>47	Median: 64<br>Median: 61
Vinik et al. 2016 (ELECT).	Lanreotide	Pancreas	No	Any (95), extrahepatic (24)<br>Any (94), extrahepatic (40)	51<br>53	Median: 56<br>Median: 57
	**Tryptophan Hydrohylase (TPH) inhibitors**					
Kulke et al. 2017 (TELESTAR).	Telotristat etiprate	Carcinoid Tumors	n.d.	n.d.<br>n.d.	46.7<br>53.3<br>44.4	Mean: 63.3<br>Mean: 62.4<br>Mean: 64.9

Pavel et al. 2018 (TELECAST).	Telotristat ethyl	Carcinoid Tumors	n.d.	n.d.	50<br>44<br>40	Mean: 62.2<br>Mean: 63.6<br>Mean: 62.7
Kulke et al. 2014	Telotristat etiprate	Carcinoid Tumors	n.d.<br>n.d.	n.d.<br>n.d.	2<br>3<br>1<br>2<br>4	Mean:62.4<br>Mean: 51.7<br>Mean:61.0<br>Mean:52.7<br>Mean:65.7
	**mTOR inhibitors**					
Pavel et al. 2017 (RADIANT-2).	Everolimus+octreotide	Pancreas, GI, lung, other	G1 (77), G2 (18), G3 (0), unknown (5)<br>G1 (82), G2 (14), G3 (0), unknown(3)	79<br>81	55<br>42	Median: 60<br>Median: 60
Yao et al. 2011 (RADIANT-3).	Everolimus	GI, Lung, other	G1 (63), G2 (37), unknown (0)<br>G1 (67), G2 (33)	0<br>0	57<br>45	Median: 65<br>Median: 60
Yao et al. 2016 (RADIANT-4).	Everolimus	GI , other	G1 (85), G2 (15)<br>G1 (84), G2 (15)	59<br>66	55<br>49	Median: 61<br>Median: 61
	**TKI**					
Raymond et al. 2011	Sunitinib	Pancreas	G1 (8), G2 (34), n.a. (58)<br>G1 (7), G2 (35), n.a. (58)	29<br>25	51<br>53	Median: 56<br>Median: 57
Wells et al. 2011 (ZETA)	Vandetanib	Medullary thyroid carcinoma	n.a.	n.d.	42<br>44	Mean: 50.7<br>Mean : 50.3
Schlumberger et al. 2017 (EXAM).	Cabozatinib	Medullary thyroid carcinoma	n.a.	n.a.	31.1<br>36.9	Mean: 54.4
Xu et al. 2020 (SANET-p).	Surufatinib	Pancreas	G1(14), G2(99)	10<br>5	47<br>53	Median: 51
Carbonero et al. 2021(Axinet)	Axinitib	Extra-pancreatic NETs	G1,G2	n.a.	48<br>48	Median:61
Bergland et al 2019	Pazopanib Hydrochloride	Carcinoid Tumors	G1	n.a.	64.9<br>44.6	Median: 63
Xu et al. 2020 (SANET-ep).	Surufatinib	Extra-pancreatic NETs	G1 (16), G2 (84)<br>G1 (16), G2 (84)	4<br>3	43<br>49	Median: 52<br>Median: 54

Abbreviations. mTOR: mechanistic target of rapamycin; NET: neuroendocrine tumor; n.a.: non available; SSAs: somatostatin analogs; TKI: tyrosine kinase inhibitor; TPH: tryptophan hydrohylase

Supplementary Table 2. Search Strategy

Search terms in MEDLINE			Number of references
Neuroendocrine Tumors			
	1	(neuroendocrine adj4 (tumo?r* OR neoplas* OR cancer* OR carcinom* OR malignanc*)).ab,kf,ti.	23,009
	2	neuroendocrine tumors/ OR adenoma, acidophil/ OR adenoma, basophil/ OR adenoma, chromophobe/ OR apudoma/ OR carcinoid tumor/ OR malignant carcinoid syndrome/ OR carcinoma, neuroendocrine/ OR somatostatinoma/ OR vipoma/ OR Multiple Endocrine Neoplasia Type 1/ OR carcinoma, medullary/ OR carcinoma, merkel cell/ OR exp neurilemmoma/ OR exp paraganglioma/ OR pheochromocytoma/	77,106
	3	((carcinom* adj2 medulla*) OR (cancer adj2 medulla*) OR (tumo?r* adj2 medulla*)).ab,kf,ti.	9,051
	4	adenoma, islet cell/ OR insulinoma/ OR carcinoma, islet cell/ OR gastrinoma/ OR glucagonoma/	9,577
	5	(pNET OR PanNEN OR PanNET OR 'Non functioning pancreatic endocrine tumo?r*' OR 'Non-functioning pancreatic endocrine tumo?r*' OR 'adenoma islet cell' OR insulinoma* OR 'carcinom* islet cell' OR gastrinoma* OR glucagonoma* OR 'Zollinger Ellison Syndrome' OR 'pancreasislet cell tumo?r*' OR 'pancreas islet cell carcinom*').ab,kf,ti.	12,836
	6	(lungcarcinoid* OR carcinoid* OR somatostatinoma* OR vipoma* OR apudoma* OR (adenoma* adj2 chromophobe)OR (adenoma* adj2 basophil*) OR (adenoma* adj2 acidophil*) OR "Multiple Endocrine Neoplasia Type 1" OR "MEN 1" OR Neurilemmoma* OR Neurilemoma* OR Schwannoma* OR Neurinoma* OR Schwannomatosis OR Schwannomatoses OR Paraganglioma* ORPheochromocytoma*).ab,kf,ti.	62,061
	7	(merk* adj4 (tumo?r* OR cancer OR carcinom*)).ab,kf,ti.	3,521
	8	("Gastro-enteropancreatic neuroendocrine tumor" OR "Thyroid cancer, medullary").mp.	1,346
	9	1 OR 2 OR 3 OR 4 OR 5 OR 6 OR 7 OR 8	123,208
Therapeutics			
	10	exp Therapeutics/ OR exp Antineoplastic Agents/	5,356,195
	11	(therap* OR radiotherap* OR radiofrequency ablat* OR radio-frequency ablat* OR radio frequency ablat* OR RFablat* OR thermoablat* OR local ablat* OR transarterial emboli?ation OR transarterial emboli?ation OR transarterial chemoemboli?ation OR transarterial chemoemboli?ation ORradioemboli?ation OR somatostatin OR telotristat OR interferon OR chemotherap* OR peptide recept OR radiotherap* OR targeted molecular therap* OR	3,507,527

		immunotherap* OR radiopeptide OR DOTATOC OR DOTATATE OR PRRT).ab,kf,ti.	
	12	Lutetium/ OR (lutetium OR edotreotide).mp.	2,133
	13	(radiopeptide* OR "peptide receptor radionuclear therapy" OR OCTREOTATE OR DOTA OR 177Lu OR Lu177 OR "Lu-177" OR "177-Lu" OR Lutetium177).ab,kf,ti.	5,430
	14	10 OR 11 OR 12 OR 13	7,381,126
	15	9 AND 14	35,499
	16	(randomized OR placebo OR randomly OR trial OR groups).ab.	2,909,354
	17	drug therapy.ab,kf,ti.	49,,229
	18	(controlled clinical trial OR randomized controlled trial).pt.	606,435
	19	16 OR 17 OR 18	3,093,740
	20	exp animals/ NOT humans.sh.	4,757,420
	21	19 NOT 20	2,643,945
Combined sets			
	22	15 AND 21	2,786
Limits: Randomized controlled trials			
	23	limit 22 to (clinical trial, phase i OR clinical trial, phase ii OR clinical trial OR case reports OR editorial OR letter OR metaanalysis OR "review" OR "systematic review")	1,167
	24	22 NOT 23	1,619
	25	limit 15 to ("in data review" OR in process OR "pubmed not medline")	3,295
	26	(random* OR group* OR trial* OR placebo OR phase III OR phase IV OR controlled).ab,kf,ti.	5,297,601
	27	25 AND 26	738
	28	limit 27 to (case reports or "review")	222
	29	27 NOT 28	516
	30	24 OR 29	1,916

Search terms in Embase			Number of references
Neuroendocrine tumors			
	1.	(neuroendocrine NEAR/4 tumo$r*):ti,ab,kw	29,104
	2.	(neuroendocrine NEAR/4 cancer*):ti,ab,kw	3,100
	3.	(neuroendocrine NEAR/4 neoplas*):ti,ab,kw	4,240
	4.	(neuroendocrine NEAR/4 carcinom*):ti,ab,kw	9,793
	5.	(neuroendocrine NEAR/4 malignanc*):ti,ab,kw	621
	6.	(adenoma* NEAR/2 acidophil):ti,ab,kw	33
	7.	(adenoma* NEAR/2 basophil):ti,ab,kw	28
	8.	(adenoma* NEAR/2 chromophobe):ti,ab,kw	590

	9.	'gastroenteropancreatic neuroendocrine tumor'/exp<br>OR 'neuroendocrine carcinoma'/exp OR 'paraganglioma'/deOR 'carotid body tumor'/de OR 'thyroid carcinoma'/de<br>OR 'bronchus carcinoid'/de OR 'carcinoid syndrome'/deOR 'gastrointestinal carcinoid'/exp<br>OR 'apudoma'/de<br>OR 'vipoma'/de OR 'somatostatinoma'/de OR 'medullarycarcinoma'/de OR 'thyroid medullary carcinoma'/de<br>OR 'neurilemoma'/de OR 'adrenal medulla tumor'/de<br>OR 'pheochromocytoma'/de OR 'neuroendocrine tumor'/deOR 'multiple endocrine neoplasia type 1'/de	128,860
	10.	lungcarcinoid*:ti,ab,kw OR carcinoid*:ti,ab,kw<br>OR somatostatinoma*:ti,ab,kw OR vipoma*:ti,ab,kw OR apudoma*:ti,ab,kw OR neurilemmoma*:ti,ab,kw OR neurilemoma*:ti,ab,kw OR schwannoma*:ti,ab,kwOR neurinoma*:ti,ab,kw OR schwannomatosis:ti,ab,kw<br>OR schwannomatoses:ti,ab,kw OR paraganglioma*:ti,ab,kwOR pheochromocytoma*:ti,ab,kw<br>OR 'multiple endocrine neoplasia type 1':ti,ab,kw OR 'men 1':ti,ab,kw OR 'pancreasislet cell tumo$r*':ti,ab,kw OR 'pancreas islet cell<br>carcinom*':ti,ab,kw	81,383
	11.	((carcinom* NEAR/2 medulla*):ti,ab,kw) OR ((cancer NEAR/2 medulla*):ti,ab,kw) OR ((tumo$r*<br>NEAR/2 medulla*):ti,ab,kw)	12,065
	12.	(merk* NEAR/4 tumo$r*):ti,ab,kw	662
	13.	(merk* NEAR/4 cancer):ti,ab,kw	241
	14.	(merk* NEAR/4 carcinom*):ti,ab,kw	4,621
	15.	'pnet':ti,ab,kw OR 'pannen':ti,ab,kw OR 'pannet':ti,ab,kw OR 'non-functioning pancreatic endocrine tumo$r*':ti,ab,kw<br>OR 'adenoma islet cell*':ti,ab,kw OR insulinoma:ti,ab,kw OR 'carcinoma islet cell*':ti,ab,kw OR gastrinoma:ti,ab,kwOR glucagonoma:ti,ab,kw OR 'zollinger ellison syndrome':ti,ab,kw	17,592
	16.	'insulinoma'/de OR 'gastrinoma'/de OR 'glucagonoma'/de<br>OR 'pancreas islet cell carcinoma'/exp OR 'pancreas islet celltumor'/exp	24,540
	17.	1 OR 2 OR 3 OR 4 OR 5 OR 6 OR 7 OR 8 OR 9 OR<br>10 OR 11 OR 12 OR 13 OR 14 OR 15 OR 16	185,410
Therapeutics			
	18.	'therapy'/de OR 'cancer therapy'/exp OR 'drug therapy'/exp OR 'nonsurgical invasive therapy'/exp OR 'radiotherapy'/exp<br>OR 'therapy effect'/exp OR 'antineoplastic agent'/exp	5,950,651
	19.	'lutetium'/de OR 'lutetium 177'/de OR 'edotreotide'/de	5,456
	20.	lutetium:ti,ab,kw OR lutetium177:ti,ab,kw<br>OR edotreotide:ti,ab,kw OR radiopeptide*:ti,ab,kw<br>OR dotatoc:ti,ab,kw OR dotatate:ti,ab,kw OR prrt:ti,ab,kw OR 'peptide receptor radionuclear therapy':ti,ab,kw	12,706

		OR octreotate:ti,ab,kw OR dota:ti,ab,kw OR 177lu:ti,ab,kw OR lu177:ti,ab,kw OR 'lu-177':ti,ab,kw OR '177-lu':ti,ab,kw	
	21.	therap* OR radiotherap* OR 'radiofrequency ablat*':ab,kw,tiOR 'radio-frequency ablat*':ab,kw,ti OR 'radio frequency ablat*':ab,kw,ti OR rfablat*:ab,kw,ti OR thermoablat*:ab,kw,ti OR 'local ablat*':ab,kw,ti OR 'transarterial emboli$ation':ab,kw,ti OR 'transarterial chemoemboli$ation':ab,kw,ti OR radioemboli$ation:ab,kw,ti OR somatostatin:ab,kw,ti OR telotristat:ab,kw,ti OR interferon:ab,kw,ti OR chemotherap*:ab,kw,ti OR 'peptide receptor radiotherap*':ab,kw,ti OR 'targeted molecular therap*':ab,kw,ti OR immunotherap*:ab,kw,ti OR radiopeptide:ab,kw,ti OR dotatoc:ab,kw,ti OR dotatate:ab,kw,ti OR prrt:ab,kw,ti	10,160,908
	22.	18 OR 19 OR 20 OR 21	11,358,892
Combined sets			
	23.	17 AND 22	80,754
Limits: Randomized controlled trials			
	24.	'drug therapy':ab,kw,ti	59,407
	25.	((single OR double OR treble OR triple) NEAR/5 (blind* OR mask OR masked)):ti,ab,kw	249,190
	26.	placebo:ti,ab,kw OR 'double blind':ti,ab,kw OR 'single blind':ti,ab,kw OR (((random* OR controlled) NEAR/5 trial*):ti,ab,kw) OR ((allocat* NEAR/5 random):ti,ab,kw)	860,099
	27.	'controlled clinical trial'/exp OR 'randomized controlled trial'/exp	802,339
	28.	24 OR 25 OR 26 OR 27	1,253,059
	29.	23 AND 28	3,820
Excluded			
	30.	([conference abstract]/lim OR [conference paper]/lim OR [conference review]/lim OR [data papers]/lim OR [editorial]/lim OR [letter]/lim OR [note]/lim OR [review]/lim OR [short survey]/lim)	
	31.	29 NOT 30	1,302

Search terms in Cochrane via Wiley			Number of references
Neuroendocrine tumors			
	1	MeSH descriptor: [Neuroendocrine Tumors] this term only	161
	2	MeSH descriptor: [Adenoma, Acidophil] this term only	0
	3	MeSH descriptor: [Adenoma, Basophil] this term only	0
	4	MeSH descriptor: [Adenoma, Chromophobe] this term only	1
	5	MeSH descriptor: [Apudoma] this term only	0
	6	MeSH descriptor: [Carcinoid Tumor] this term only	85
	7	MeSH descriptor: [Malignant Carcinoid Syndrome] this term only	41
	8	MeSH descriptor: [Carcinoma, Neuroendocrine] this term only	50
	9	MeSH descriptor: [Somatostatinoma] this term only	3
	10	MeSH descriptor: [Vipoma] this term only	2
	11	MeSH descriptor: [Multiple Endocrine Neoplasia Type 1] this term only	8
	12	MeSH descriptor: [Carcinoma, Medullary] this term only	15
	13	MeSH descriptor: [Carcinoma, Merkel Cell] this term only	17
	14	MeSH descriptor: [Neurilemmoma] explode all trees	73
	15	MeSH descriptor: [Paraganglioma] explode all trees	64
	16	MeSH descriptor: [Pheochromocytoma] this term only	61
	17	MeSH descriptor: [Adenoma, Islet Cell] this term only	27
	18	MeSH descriptor: [Insulinoma] this term only	11
	19	MeSH descriptor: [Carcinoma, Islet Cell] this term only	7
	20	MeSH descriptor: [Gastrinoma] this term only	6
	21	MeSH descriptor: [Glucagonoma] this term only	2
	22	1 OR 2 OR 3 OR 4 OR 5 OR 6 OR 7 OR 8 OR 9 OR 10 OR 11 OR 12 OR 13 OR 14 OR 15 OR 16 OR 17 OR 18 OR 19 OR 20 OR 21	455
	23	(neuroendocrine NEAR/4 (tumo?r* OR neoplas* OR cancer* OR carcinom*OR malignanc*)):ti,ab,kw	963
	24	(((carcinom* NEAR/2 medulla*) OR (cancer NEAR/2 medulla*) OR (tumo?r* NEAR/2 medulla*))):ti,ab,kw	182
	25	(pNET OR PanNEN OR PanNET OR 'Non functioning pancreatic endocrine tumo?r*' OR 'Non-functioning pancreatic endocrine tumo?r*' OR 'adenoma islet cell' OR insulinoma* OR 'carcinom* islet cell' OR gastrinoma* OR glucagonoma* OR 'Zollinger Ellison Syndrome' OR 'pancreas islet cell tumo?r*' OR 'pancreas islet cell carcinom*') :ti,ab,kw	710

	26	(lungcarcinoid* OR carcinoid* OR somatostatinoma* OR vipoma* OR apudoma* OR (adenoma* NEAR/2 chromophobe) OR (adenoma* NEAR/2 basophil*) OR (adenoma* NEAR/2 acidophil*) OR "Multiple Endocrine Neoplasia Type 1" OR "MEN 1" OR Neurilemmoma* OR Neurilemoma* OR Schwannoma* OR Neurinoma* OR Schwannomatosis OR Schwannomatoses OR Paraganglioma* OR Pheochromocytoma*):ti,ab,kw	951
	27	((merk* NEAR/4 (tumo?r* OR cancer OR carcinom*))):ti,ab,kw	77
	28	23 OR 24 OR 25 OR 26 OR 27	2,440
	29	22 OR 28	2,464
Therapeutics			
	30	MeSH descriptor: [Therapeutics] explode all trees	304,349
	31	MeSH descriptor: [Antineoplastic Agents] explode all trees	12,136
	32	MeSH descriptor: [Lutetium] this term only	2
	33	30 OR 31 OR 32	307,790
	34	(therap* OR radiotherap* OR 'radiofrequency ablat*' OR 'radio-frequency ablat*' OR 'radio frequency ablat*' OR RFablat* OR thermoablat* OR 'local ablat*' OR 'transarterial emboli?ation' OR 'transarterial emboli?ation' OR 'transarterial chemoemboli?ation' OR 'transarterial chemoemboli?ation' OR radioemboli?ation OR somatostatin OR telotristat ORinterferon OR chemotherap* OR 'peptide recept OR radiotherap*' OR 'targeted molecular therap*' OR immunotherap* OR radiopeptide OR DOTATOC OR DOTATATE OR PRRT):ti,ab,kw	785,854
	35	(lutetium OR edotreotide OR radiopeptide* OR "peptide recept OR radionuclear therapy" OR OCTREOTATE OR DOTAOR 177Lu OR Lu177 OR "Lu-177" OR "177-Lu" OR lutetium177):ti,ab,kw	261
	36	34 OR 35	785,903
	37	33 OR 36	857,235
Combined sets			
	38	29 AND 37	1,664
	39	29 AND 37 in Trials	1,643

Search terms in Clinical trials.gov			Number of references
Neuroendocrine tumors			
	1	Recruiting, Completed, Suspended, Terminated, Withdrawn, Unknown status Studies | Interventional Studies | Neuroendocrine Tumors | Antineoplastic Agents | Phase 3, 4)	122
	2	Recruiting, Completed, Suspended, Terminated, Withdrawn, Unknown status Studies | Interventional Studies | Neuroendocrine Tumors | Therapy | Phase 3, 4	120
	3	Radiotherapy | Recruiting, Completed, Suspended,Terminated, Withdrawn, Unknown status Studies | Interventional Studies | Neuroendocrine Tumors | Phase 3, 4	28
	4	Recruiting, Completed, Suspended, Terminated, Withdrawn, Unknown status Studies | Interventional Studies | Neuroendocrine Tumors | Drug Therapy | Phase 3, 4	66
	5	Recruiting, Completed, Suspended, Terminated, Withdrawn, Unknown status Studies | Interventional Studies | Neuroendocrine Tumors | Drug | Phase 3, 4	167
Neuroendocrine carcinoma			
	7	Recruiting, Completed, Suspended, Terminated, Withdrawn, Unknown status Studies | Interventional Studies | Neuroendocrine Carcinoma | Antineoplastic Agents | Phase 3, 4	2
	8	Recruiting, Completed, Suspended, Terminated, Withdrawn, Unknown status Studies | Interventional Studies |Neuroendocrine Carcinoma | Therapy | Phase 3, 4	4
	9	Recruiting, Completed, Suspended, Terminated, Withdrawn, Unknown status Studies | Interventional Studies | Neuroendocrine Carcinoma | Therapeutics | Phase 3, 4	3
	10	Recruiting, Completed, Suspended, Terminated, Withdrawn, Unknown status Studies | Interventional Studies | Neuroendocrine Carcinoma | Radiotherapy| Phase 3, 4	0
	11	Recruiting, Completed, Suspended, Terminated, Withdrawn, Unknown status Studies | Interventional Studies | Neuroendocrine Carcinoma | Drug Therapy | Phase 3, 4	1
	12	Recruiting, Completed, Suspended, Terminated, Withdrawn, Unknown status Studies | Interventional Studies | Neuroendocrine Carcinoma | Drug | Phase 3, 4	5
		1-12/OR	518

Supplementary Table 3: A. List of serious and not-serious disorders B. Specific cardiac adverse effects and C. Specific vascular adverse effects including hypertension assessed in the present study in order to categorize toxicities into different safety profiles (all grade toxicities and grade 3 and 4 toxicities) according to the National Cancer Institute Common Terminology Criteria for Adverse Events v5.0 mainly from clinicaltrials.gov.

A. Blood and lymphatic system disorders; Cardiac disorders; Ear and labyrinth disorders; Endocrine disorders; Gastrointestinal disorders; General disorders; Hepatobiliary disorders; Immune system disorders; Infections and infestations; Injury, poisoning and procedural complications; Metabolism and nutrition disorders; Musculoskeletal and connective tissue disorders; Neoplasms benign, malignant and unspecified; Nervous system disorders; Psychiatric disorders; Renal and urinary disorders; Reproductive system and breast disorders; Respiratory, thoracic and mediastinal disorders; Vascular disorders.

B. Cardiac disorders: acute coronary syndrome, angina pectoris, coronary artery stenosis, aortic valve stenosis, pulmonary valve stenosis, cardiac failure, acute cardiac failure, congestive cardiac failure, cardiopulmonary failure, ventricular failure, tricuspid valve incompetence, mitral valve incompetence, left ventricular dysfunction , right ventricular dysfunction , atrioventricular block second degree, myocardial infarction, myocardial dysfunction, myocarditis, pericarditis, pericardial effusion, pericardial haemorrhage, palpitations, tachycardia paroxysmal , cardiac arrest, cardio-respiratory arrest, Acute myocardial infarction, arrythmia, supraventricular tachycardia, ventricular arrhytmia, atrial fibrillation, atrial flutter, bradycardia.

C. Vascular disorders: hypertension, hypotension, circulatory collapse, hypertensive crisis, deep vein thrombosis, vena cava thrombosis, arterial thrombosis, thromboembolic event, phlebitis, haemorhage.

© 2021 by the authors. Licensee MDPI, Basel, Switzerland. This article is an open access article distributed under the terms and conditions of the Creative Commons Attribution (CC BY) license (http://creativecommons.org/licenses/by/4.0/).
